# Correction: Modeling Hawaiian Ecosystem Degradation due to Invasive Plants under Current and Future Climates

**DOI:** 10.1371/journal.pone.0102400

**Published:** 2014-07-03

**Authors:** The PLOS ONE Staff

## Notice of Republication

This article was republished on June 3, 2014, to replace Files S1-S18, which had file types unsupported by the PLOS website. The publisher apologizes for the issue. Please view this article again to download the files in ZIP format. The originally published, uncorrected article and the republished, corrected article are provided here for reference.

## Supporting Information

File S1 Originally published, uncorrected article.
(PDF)

File S2 Republished, corrected article.
(PDF)
