# Supplementary material for: Correction: Modeling Hawaiian Ecosystem Degradation due to Invasive Plants under Current and Future Climates
Source: PLoS One. 2014 Jul 3;9(7):e102400. doi: 10.1371/journal.pone.0102400 (PMC4081720; doi:10.1371/journal.pone.0102400)
Supplement: File S1 — Originally published, uncorrected article. (PDF) [file pone.0102400.s001.pdf]

PLOS | ONE

Modeling Hawaiian Ecosystem Degradation due to Invasive Plants under Current and Future Climates

Adam E. Vorsino[1]*, Lucas B. Fortini[2,3], Fred A. Amidon[1], Stephen E. Miller[1], James D. Jacobi[2], Jonathan P. Price[4], Sam 'Ohukani'ohi'a Gon III[5], Gregory A. Koob[6]

1 Strategic Habitat Conservation Division, Pacific Islands Office, United States Fish & Wildlife Service, Honolulu, Hawaii, United States of America, 2 Pacific Island Ecosystems Research Center, United States Geological Survey, Honolulu, Hawaii, United States of America, 3 Pacific Islands Climate Change Cooperative, Honolulu, Hawaii, United States of America, 4 Department of Geography, University of Hawaii at Hilo, Hilo, Hawaii, United States of America, 5 The Nature Conservancy of Hawaii, Honolulu, Hawaii, United States of America, 6 Natural Resources Conservation Service, United States Department of Agriculture, Honolulu, Hawaii, United States of America

Abstract

Occupation of native ecosystems by invasive plant species alters their structure and/or function. In Hawaii, a subset of introduced plants is regarded as extremely harmful due to competitive ability, ecosystem modification, and biogeochemical habitat degradation. By controlling this subset of highly invasive ecosystem modifiers, conservation managers could significantly reduce native ecosystem degradation. To assess the invasibility of vulnerable native ecosystems, we selected a proxy subset of these invasive plants and developed robust ensemble species distribution models to define their respective potential distributions. The combinations of all species models using both binary and continuous habitat suitability projections resulted in estimates of species richness and diversity that were subsequently used to define an invasibility metric. The invasibility metric was defined from species distribution models with <0.7 niche overlap (Warrens *I*) and relatively discriminative distributions (Area Under the Curve >0.8; True Skill Statistic >0.75) as evaluated per species. Invasibility was further projected onto a 2100 Hawaii regional climate change scenario to assess the change in potential habitat degradation. The distribution defined by the invasibility metric delineates areas of known and potential invasibility under current climate conditions and, when projected into the future, estimates potential reductions in native ecosystem extent due to climate-driven invasive incursion. We have provided the code used to develop these metrics to facilitate their wider use (Code S1). This work will help determine the vulnerability of native-dominated ecosystems to the combined threats of climate change and invasive species, and thus help prioritize ecosystem and species management actions.

Citation: Vorsino AE, Fortini LB, Amidon FA, Miller SE, Jacobi JD, et al. (2014) Modeling Hawaiian Ecosystem Degradation due to Invasive Plants under Current and Future Climates. PLoS ONE 9(5): e95427. doi:10.1371/journal.pone.0095427

Editor: Paul Adam, University of New South Wales, Australia

Received August 9, 2013; **Accepted** March 26, 2014; **Published** May 7, 2014

This is an open-access article, free of all copyright, and may be freely reproduced, distributed, transmitted, modified, built upon, or otherwise used by anyone for any lawful purpose. The work is made available under the Creative Commons CC0 public domain dedication.

Funding: This work was funded by the United States Fish and Wildlife Service. Employees of the Fish and Wildlife Service analyzed the data, prepared the manuscript, and decided to publish in this journal.

Competing Interests: The authors have declared that no competing interests exist.

* E-mail: Adam_Vorsino@fws.gov

Introduction

Occupation of native ecosystems by ecosystem-modifying invasive plants (EMIP) has been linked to changes in native biodiversity [1,2], biogeochemical heterogeneity [3–5] and ecosystem services [2]. These invaders alter the structure and/or function of the native ecosystems through both habitat degradation, and the development of non-analog communities/ecosystems [6–8]. As detailed in Vitousek *et al.* [8] and Hughes *et al.* [9], EMIPs both singly and collectively, have the potential to overrun and fragment habitat previously characterized by unique and diverse endemic communities. Although some ecosystems are resilient[10,11], continuous degradation of habitat without remediation may alter these diverse native communities to a point beyond recovery.

Long-term climate change impacts may influence native ecosystem resilience as well as shift the distribution of EMIPs [12–15]. For instance, Willis *et al.* [12] demonstrated a dramatic difference in the response of invasive species to climate change, where invasive species were found to be far better at tracking seasonal temperature changes than native and non-native non-invasive species. In the absence of management, habitat degradation by EMIPs under climate change may occur through an increase in invasive species spread, which may increase EMIP diversity (due to a redistribution of ecosystem dominance brought about by climate change [16]), and/or an increase in EMIP density. Increases in EMIP diversity and density add pressures to native ecosystems [13,16], which in combination with a reduction in ecosystem resilience may lead to an expansion of degraded habitat and increasingly diminish endemic diversity [16–18]. Although there are biome- and scale-based differences in the resistance of native ecosystems to invasion [18–20], the overall trend of native contraction following EMIP incursion is consistent [21,22]. Therefore, understanding the realized and potential distribution of these EMIPs, especially in relation to climate change, is an important step in defining native ecosystem susceptibility and managing EMIP impact.

The term *invasibility* is commonly used to describe the susceptibility of native ecosystems to colonization and thus modification [23,24]. Metrics defining invasibility risk reflect habitat suitability in both invaded and unoccupied habitat. In order to understand consistent and continuous ecosystem degradation by these EMIPs under climate change a landscape level

Table 1. Hawaiian Ecosystem Modifying Invasive Plants (EMIP's) selected for this analysis.

Species Name	CommonName	[3]Code	Family	Established in Hawaii	[1]Habitat (Wagner *et al*, [43])	[2]Habitat (Price *et al*, [42])	Number of Records	[4]RA	RA Score[2]
Clidemia hirta	Koster's curse	CliHir	Melastomataceae	1941 (72 years)	Mesic to Wet Forest	Mesic-Wet	60	High Risk	27
Falcataria moluccana	Albizia	FalMol	Fabaceae	1920 (93 years)	Disturbed Mesic to Wet Areas	Mesic	414	High Risk	8
Hedychium gardnerianum	Kāhili ginger	HedGar	Zingiberaceae	1940 (73 years)	Wet Forest	Wet	218	High Risk	16
Lantana camara	Lantana	LanCam	Verbenaceae	1858 (155 years)	-	Dry-Mesic	307	High Risk	32
Leucaena leucocephala	Koa haole, ēkoa	LeuLeu	Fabaceae	1837 (176 years)	-	Dry	502	High Risk	15
Melinis minutiflora	Molasses grass	MelMin	Poaceae	1910 (103 years)	Dry to Mesic Areas	Mesic	298	High Risk	18
Miconia calvescens	Miconia	MicCal	Melastomataceae	~1983 (30 years)	-	Mesic-Wet	102,595	High Risk	14
Morella faya	Firetree	MorFay	Myricaceae	1926 (87 years)	Mesic to Wet Forest	Mesic	968	High Risk	17
Panicum maximum	Guinea grass	PanMax	Poaceae	1871 (142 years)	-	Dry	44	High Risk	17
Passiflora tarminiana	Banana poka	PasTar	Passifloraceae	1926 (87 years)	Mesic Forests	Dry	5857	High Risk	24
Pennisetum clandestinum	Kikuyu grass	PenCla	Poaceae	1923 (90 years)	Dry to Mesic Forests	Mesic	135	High Risk	18
Pennisetum setaceum	Fountain grass	PenSet	Poaceae	1914 (99 years)	Dry/Open Areas (e.g. Lava fields)	Dry	317	High Risk	26
Psidium cattleianum	Strawberry guava	PsiCat	Myrtaceae	1825 (188 years)	Mesic and Wet Forest	Wet	540	High Risk	18
Schinus terebinthifolius	Christmas berry	SchTer	Anacardiaceae	1911 (102 years)	Disturbed Mesic Forests	Mesic	916	High Risk	19
Setaria palmifolia	Palmgrass	SetPal	Poaceae	1903 (110 years)	Mesic to Wet Forest	Wet	1059	High Risk	7**
Sphaeropteris (syn: Cyathea) cooperi	Australian tree fern	SphCoo	Cyatheaceae	1950 (63 years)	Wet Forest	Dry	79	High Risk	8
Ulex europaeus	Gorse	UleEur	Fabaceae	1914 (99 years)	-	Mesic	473	High Risk	20
			**Avg. Establishment:**	**~1908 (104±41 years)**		**SUM:**	**114,782**	**Avg. RA**	**18.8**

[1]Habitat is described by a broad habitat descriptor defining the ecotype and year naturalized (or first collected) as inferred from [43,122].
[2]The moistures index developed by Price *et al*. [42] defined species specific moisture types from collection data.
[3]For ease of interpretation each species was coded using a representative six letter combination.
[4]Risk Assessment (RA) scores and categories defined by Dahler *et al*. [19] and Pheloung *et al*. [41] accessed from: http://www.hear.org/pier/index.htm.
**Based on estimates for Australia [41] accessed from: http://www.hear.org/pier/wra/australia/sepal-wra.htm.
doi:10.1371/journal.pone.0095427.t001

analysis is necessary. One such landscape based approach, species distribution modeling (SDM), has been used extensively to understand both single and multi-species (i.e. richness/diversity) distributions in both natural and invaded landscapes [25–27]. The multitude of SDM methodologies all have the ability (with varying accuracy) to both define and predict the theorized realized niche of an organism (based on biotic and abiotic variables), and project that habitat onto specific climate change scenarios [26,28,29]. By combining these niche estimates for multiple species, conservationists and ecologists can predict and project hotspots of native and non-native species richness and diversity [27,28,30,31]. These types of landscape-based predictive analyses offer a powerful tool to predict actual and potential habitat degradation in relation to non-native species invasion. Delineating non-native hotspots allows ecosystem managers to understand the distribution of currently invaded habitat, as well as recognize the invasive organisms' potential distribution (i.e. invasibility) [30,32,33]. Recognizing the invader's current and potential distributions will enable ecosystem mangers and regulators to prioritize resources and identify native dominated habitat with a high likelihood of EMIP incursion [30,31].

Hawaii, an isolated archipelago extolled for the biodiversity of its endemic flora and fauna, also has the highest number of endangered species in the United States [34]. Of the 919 plants federally listed as threatened or endangered in the United States, approximately 400 (~43%) of them occur in Hawaii [35]. Many of the habitats these plants once flourished in are now dominated by highly invasive EMIPs [6,34], which dramatically alter community structure and ecosystem processes [36–38]. It has been estimated that of the ~8–10,000 plant taxa introduced to the Hawaiian islands only ~90 are regarded as extremely harmful due to competitive ability, ecosystem modification, and/or biogeochemical habitat degradation [39]. Given that non-native/invasive species that have become naturalized make up ~ ½ of all species in Hawaii [40], the small number of highly invasive EMIPs is somewhat surprising, and potentially encouraging, as controlling this small group of highly invasive ecosystem modifiers could significantly reduce native ecosystem degradation.

For this study, we compiled data for the top 17 Hawaii-based EMIPs, characterized their distributions and developed a novel metric attempting to describe overall invasibility within Hawaii. The distribution of each invasive plant species was modeled using an ensemble of SDM methodologies to project the distribution of actual and potential degraded habitat, and assess whether modeled EMIP richness and diversity can be used as a proxy of invasibility. We then estimated the current (2013) and future (~2100) distribution of invasibility (and its change over time) throughout Hawaii, and in relation to federally designated critical habitat for endemic Hawaiian organisms listed as threatened or endangered. By characterizing invasibility over a geographic landscape, a discriminative distribution of invasive species diversity and hotspots [30] was developed which can be used to explore island biogeographic relationships, and pro-actively manage current and future invasive incursion.

Materials and Methods

A. Invasive Species Occurrence Data

A set of 17 EMIPs were selected for this analysis based on high risk assessment scores estimated by Dahler *et al.* [19], Pheloung *et al.* [41] (Table 1), expert opinion of risk to native ecosystems and data availability. The distributions of these invasive plants encompass a broad range of regional characteristics as determined by a Hawaii specific moisture index [42] overlaid with collection localities and verified by Wagner et al. [43] (Table 1). A six letter acronym, using the first three letters of both the genus and species names, is used henceforth to code species names (Table 1).

A total of 114,782 location records for all EMIPs were collected across the main Hawaiian islands (Kauai, Oahu Molokai, Maui, Lanai, Hawaii) by the Kauai, Oahu, Maui, and Hawaii Island Invasive Species Councils, the United States National Park Service and the U. S. Geological Survey (see Fig. 1). The data included location information from both managed (complete or partial removal of the invasive plant) and unmanaged sites. All location records were used to define and project the SDMs because treated and untreated sites were both occupied by the species. The number of occurrences collected per species (Table 1) varied greatly because collections were dependent on the management priorities of the respective collecting organizations, thus, density or number of collections are not necessarily a correlate of invasive risk or degree of establishment.

When using an SDM approach it is assumed that the species is in equilibrium with environmental covariates used to derive that distribution over the defined landscape [44]. Violating this equilibrium assumption produces a model that is constrained by the location of occurrences at the time of collection. Although many SDM methodologies are relatively robust to violations of environmental equilibrium, using data limited to the onset of invasion may skew the model prediction to a small subset of an invasive organism's suitable habitat (increasing commission error). Some studies have attempted to circumvent non-equilibrium by using both natal and invaded habitat for invaded habitat projections [33,45], however this may also underestimate the potential range of the species within the novel habitat due to probable niche expansion [44,46–48]. In an attempt to address violations of equilibrium due to recent invasions, Vaclavik *et al.* [44] suggested that invasive habitat suitability projections should be conducted as the species tends closer to equilibrium. To address these concerns establishment dates for all species selected were reviewed to assess environmental equilibrium of the EMIPs in Hawaii (Table 1). Of all the species selected, the most extensively collected (and distributed) was also the most recently established (e.g. *MicCal*). Even while accounting for the more recent invasion of *MicCal*, the 17 invasive species selected for this study had an average establishment time of 104 years (SD ± 40 years). We feel that the selected species have had sufficient time to establish since introduction and are at or near equilibrium with the current environment.

B. Environmental Indices

A total of 24 continuous abiotic environmental indices, including 19 bioclimatic and 5 topographic variables, were initially considered for modeling (Fig. 2). These indices were defined for six of the main eight Hawaiian islands (Kauai, Oahu, Molokai, Maui, Lanai, and Hawaii.). All current bioclimatic variables were defined from 250 meter(m) monthly average rainfall estimates developed by Giambelluca *et al.* [49] and 500 m scaled average monthly minimum and maximum temperature maps from the PRISM Climate Group [50,51]. As with all other analyses and modeling presented, we calculated bioclimatic variables using the R statistical environment [52]. The R package 'dismo' provided methods for bioclimatic variable generation based on rainfall and temperature data [53].

Topographic variables were derived from a 30 m Digital Elevation Model as upscaled from the 10 m National Elevation Dataset (NED) [54]. These topographic variables were calculated using the R statistical environment package 'raster' [55]. All topographic variables were assumed to be biologically significant

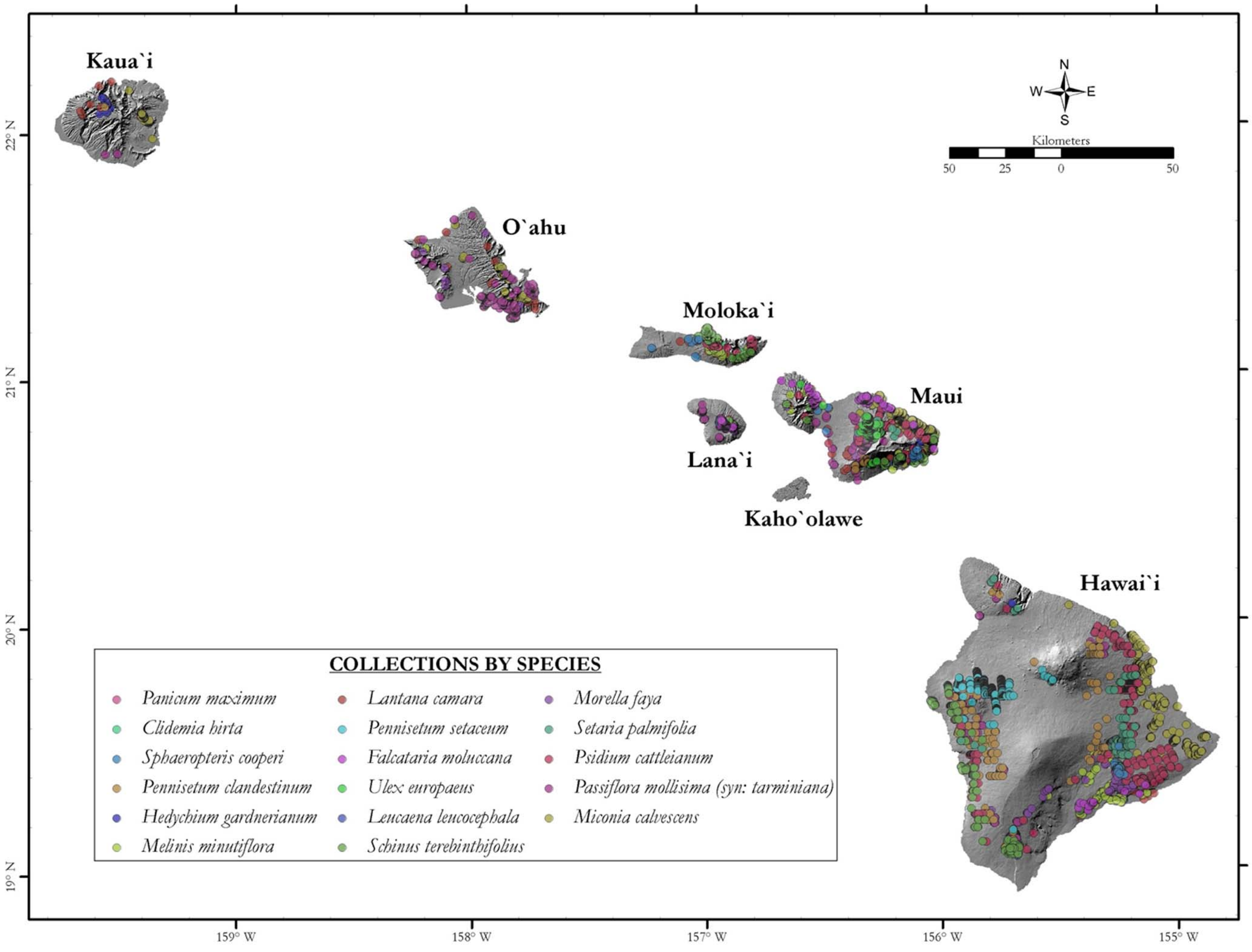

Figure 1. Collection locations for the 17 invasive species analyzed.
doi:10.1371/journal.pone.0095427.g001

to all plant species modeled [56–58] (see Fig. 2 for topographic and bioclimatic variable definitions).

Correlations between all pairs of topographic and bioclimatic variables were estimated for the complete extent of the Hawaiian Islands at 250 m resolution using a Pearson correlation coefficient (Fig. 2). These correlations were analyzed in 'raster' and plotted using 'corrplot', a graphical correlation matrix plotting package in R [59]. Multi-colinearity [57] was minimized by selecting five bioclimatic and two topographic variables with low correlation coefficients (<0.63) that were biologically relevant for SDM analyses (Table 2). These contemporary projections were used to define the current (baseline) distribution of each organism as modeled from the compiled locality data (Fig. 1).

We derived future climate projections by adding the projected change between 1990–2010 and 2080–2100 climate simulations to the baseline (non-modeled) climate data dynamic downscaled climate projections developed from the Hawaiian regional climate model (3 km^2 spatial resolution) by the International Pacific Research Center [60]. The Hawaii regional climate model is based on the Weather Research and Forecasting model V3.3 and uses the Special Report: Emissions Scenarios A1B scenario [61]and the mean of multiple Coupled Model Inter-comparison Project 3 (commonly referred to as CMIP3) global circulation models to project future climate that best represent regional climate features such as the trade wind inversion.

C. Models

a. SDM Input and Settings. Three presence-only machine learning SDM methodologies were used to model the distribution of occurrence localities over geographic space, as defined by the seven abiotic covariates described above. The three methodologies MAXENT [62], Random Forest (RF) [63] and Gradient Boosting Model (GBM) [64] were selected based on their published predictive accuracy [65–68]. MAXENT is a popular SDM tool that uses the maximum entropy approach to model species distributions by comparing the projected distribution of occurrence localities, as projected over the environmental covariates, to a null distribution (as defined by pseudo-absences) of the covariates [69]. Random Forest is a tree learning methodology modified from the bootstrap aggregation approach that builds a consensus tree from the average of a large number of de-correlated classification trees [68]. A GBM is a powerful classification tree learning methodology that attempts to improve the predictive accuracy of decision trees through boosting. The GBM approach produces a predictive classification model built from an ensemble of weaker models using an additive expansion approach that builds successive classification trees in an *a priori* manner [68,70].

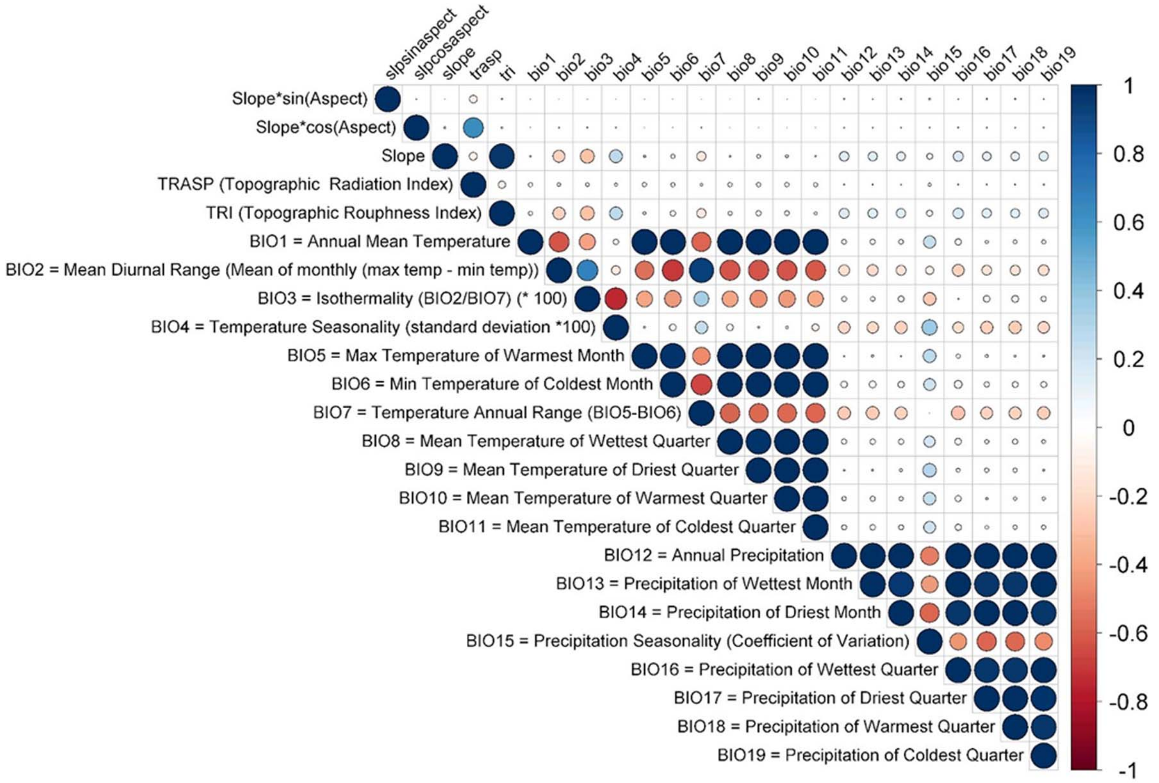

Figure 2. Pearson correlation coefficient matrix comparing paired environmental covariates. Negative correlations are shaded red; positive correlations are shaded green. Strength of the correlation is indicated by dot size and red or green color saturation. High correlation between covariates is also indicated by the size of the colored oval delineating each comparison. The definition of each covariate (y-axis) and its coded counterpart (upper x-axis) are defined per comparison.
doi:10.1371/journal.pone.0095427.g002

All analyses were run in R using the 'biomod2' package [71]. Biomod2 is a species distribution modeling platform developed for single and multi-species SDM in which multi-model ensemble modeling, calibration, forecasting and statistical analyses can be conducted iteratively.

All analyses were projected over six of the main Hawaiian islands (Fig. 1). Initial input data consisted of presence data per species, pseudo-absence data, and the abiotic climatic and topographic variables. Since presence-only species distribution modeling relies on a Boolean definition of presence rather than density, all overlapping presence points within a pre-specified grid cell, as defined by the abiotic variable raster files (i.e. 250 m×250 m), were removed such that only a single presence point per grid cell was used.

Invasive species data collection by most (if not all) of the listed organizations mainly occur in areas of high conservation value. Given that all species modeled are of concern to each organization, overlapping collections of species occurred often. Using this inherent collection bias, in concert with the overlapping collection (or not) of multiple species, we selected pseudo-absences that would help remove the spatially auto-correlated collection bias towards high value conservation areas using the methodology of Phillips *et al.* [72]. Because occurrence data for every species modeled was collected in a relatively similar way and/or area by

Table 2. The seven bioclimatic and topographic variables used for each species distribution model.

[1]Code	Covariate Definition	[2]Representative Publications
**Bio1**	Average Annual Temperature	[15,26,123,124]
**Bio2**	Mean Diurnal Range (Mean of monthly (max temp - min temp))	[15,26]
**Bio4**	Temperature Seasonality (standard deviation *100)	[15,26,123]
**Bio12**	Mean Monthly Annual Precipitation	[15,123–125]
**Bio15**	Precipitation Seasonality (Coefficient of Variation)	[15,123–125]
**TRI**	Topographic Roughness Index	[50,126,127]
**TRASP**	Topographic Radiation Index	[56,128]

[1]The variable code used in the text.
[2]Publications representative of the significance of the variables to plant distributions.
doi:10.1371/journal.pone.0095427.t002

each organization, the presences of all other species (while excluding the species being modeled) was used to define the pseudo-absence data, and thus define the collection background. This approach may help account for collection distributions not at equilibrium with the actual species distribution [73], although presence-only methodologies are relatively robust to violations of equilibrium especially for relatively well established invasive species [44,74]. As in the presence data, only a single point per grid cell was used to define the collection background (e.g. pseudo-absence defined background).

Many of the default settings, as specified for the specific modeling methodologies, were used and defined directly in 'biomod2'. Initial modeling options were set such that the GBM and RF analyses used 100 trees with 5 cross validation folds, whereas the maximum number of iterations in MAXENT was set to 100. For each modeled species, we further specified 500 Markov Chain randomization evaluation runs for each modeling methodology, and used a 20/80 (test/train) data split such that 20% of the presence data was used for model evaluation and 80% was used to calibrate each model. A sensitivity equals specificity threshold, as recommended by Liu *et al.* [75], was used to infer locations of likely presence/absence for all binary model projections because these model projections were used to calculate estimates of EMIP diversity.

b. Ensemble Model. All three SDM modeling approaches were then combined using an ensemble model (EM) to assess model congruence, and improve model accuracy. All EMs were developed in 'biomod2' [71]. An evaluation metric quality threshold of 0.5 was used to define the minimum scores of each models Receiver Operating Characteristic/Area Under the Curve (AUC) value (see *Model Validation Statistics*). Values above 0.5 were used in the final ensemble. An AUC evaluation metric of 0.5 corresponds to a discriminatory power no better than random [71]. Because multiple uncertainty measures were used to help infer model utility and accuracy, we felt that this threshold was sufficient to develop an accurate EM.

For each individual species ensemble we report two ensemble modeling outputs; the weighted mean probability of occurrence and committee averaging. The weighted mean probability of occurrence is similar to a standard model mean in that they both define the mean prediction of all models developed for the analysis above the quality threshold evaluation metric. However, the weighted mean probability of occurrence metric weights each model according to the evaluation metric (the higher the metric the greater the weight given to the model). The committee averaging EM is both a predictive distribution model and a measure of uncertainty. It uses the thresholded binary prediction across all models to predict presence (1) or absence (0). Locations with numbers between 0 and 1 show a ratio of uncertainty in defining the EM presence/absence.

c. Model Validation Statistics and Variable Importance. Each model was also evaluated using two commonly used SDM validation indices; the AUC, and the True Skill Statistic (TSS). The AUC validation statistic is a commonly used threshold independent accuracy index that ranges from 0 to 1 (1 = highly accurate prediction). The AUC index defines the probability that an SDM will rank a presence locality higher than an absence (here a pseudo-absence) [76]. The TSS statistic ranges from −1 to +1 and tests the agreement between the expected and observed distribution, and whether that outcome would be predicted under chance alone [76,77]. A TSS value of +1 is considered perfect agreement between the observed and expected distributions, whereas a value <0 defines a model which has a predictive performance no better than random [76,77]. The TSS statistic is very closely related to Cohens Kappa statistic (KAPPA), in that it also ranges from −1 to +1 and defines accuracy in comparison to chance, but unlike KAPPA, TSS is not affected by prevalence [77]. As recommended by Franklin *et al.* [78] and Elith *et al.* [79] multiple test statistics were used to allow a more robust assessment of model performance and validate model responses.

To understand a variable's relative importance to each model, species specific response plots and variable importance boxplots were developed per SDM in 'biomod2' using the methodologies of Elith *et al.* [80]. Response plots were developed for each variable that define the sensitivity of the prediction to variation in a single covariate while all other covariates were held constant. Using these plots allow inference into the models' ecological sensibility and significance of each variable to the organisms distribution [80].

d. Niche Overlap. We used the niche overlap metric, I, [81] to calculate pairwise niche overlap between the developed models. We selected the I statistic as an appropriate overlap metric because it makes no biological assumptions regarding habitat use and thus is more appropriate for presence-only SDM analyses [81]. The I statistic sums pair-wise differences between two SDMs to quantify niche overlap on a 0 to 1 scale, where 0 indicates no niche overlap and 1 indicates complete overlap. Niche overlap was further analyzed using an Equivalency Test [81] to assess whether EMIP SDM overlap is significantly different from that of a model developed from a random subset of both sets of occurrence localities. The test is used to assess significance of I by comparing the I similarity metric to a one-tailed normalized null distribution as defined by a random subset of compiled species locality information [81,82]. As recommended by Warren *et al.* [81], the equivalency test was replicated 100 times.

e. Projected Diversity Indices. To identify sites with ecosystems suitable for many exotic species a compilation of 17 EMs (one per species) was used. This suite of EMs was used as an "*invasibility index*" (i.e. invasibility) because it highlights areas of both actual and potential habitat degradation due to invasive species habitat suitability.

To define invasibility we first rescaled all individual SDM EMs on a scale of 0 (lowest habitat suitability) to 1 (highest habitat suitability) using an approach similar to that of Mateo *et al.* [83]. The methodology was used for both current and future SDMs, and is an estimate of potential species richness (alpha diversity) [83]. Because these species richness measures are threshold independent (i.e. they don't necessarily account for the probability of actual presence) they may overestimate richness and thus potential invasibility/degradation in certain areas [28,83]. We attempted to account for this by adapting the Shannon's Diversity Index (H) to an SDM approach to derive a threshold dependent estimate of diversity, as well as assess important information regarding species rarity/commonness [84]. Pineda *et al.* [28] used a similar threshold dependent approach to project species richness, but because they used a set of arbitrarily fixed thresholds to define presence, this likely increased omission and commission errors [29,75]. Since this approach uses a data driven threshold (i.e. equal sensitivity and specificity), it reduces the omission/commission errors associated with such methodologies [28,75]. The two major assumptions inherent to the application of H (and projected species richness) to an SDM approach are that habitat suitability is correlated with abundance, and that the thresholded Boolean presence/absence maps accurately define, and project, species presence.

To develop this threshold-dependent measure (i.e. H) we first needed to estimate the abundance of each organism (i) relative to the total abundance of ith organisms as estimated over the same geographic region; in H this estimate is defined as p_i [84]. To

estimate H for an SDM we first transformed the Boolean estimate of species presence (B) for each species so that all undefined pixel values were replaced with 0 to allow raster multiplication and define only areas predicted to have species present. We then multiplied B by the scaled suitability model for each species ($\boldsymbol{s}$) all of which were divided by the sum of the product of all species s multiplied by B. These were defined per species to create the H parameter p_i (*equation 1*). To define H, all p_i raster layers needed to again be reclassified such that all 0 s were undefined, this allowed for summation of probabilities over only species present in a certain location. The H equation, as adapted from Colwell [84], was then applied (*equation 2*).

$$p_i = (B_i \times s_i) / (B_i \sum_{i=1}^{n} s_i) \tag{1}$$

$$H = -\sum_{i=1}^{n} p_i \ln(p_i) \tag{2}$$

The Shannon Evenness Index (E) was also calculated. The E standardizes abundance of species across the geographic range of the organisms and thus allows for a better comparison between communities/pixels [85]. The E was adapted to an SDM approach by calculating the total number of species (S) to be representative of the total number of species across the extent of the analysis. The summation of B_i for all organisms was used to calculate S (*equation 3*), which was subsequently applied to the calculation of E adapted from Burton *et al.* [86] (*equation 4*).

$$S = \sum_{i=1}^{n} B_i \tag{3}$$

$$E = \frac{H}{\ln(S)} \tag{4}$$

The change in E, or Evenness Delta (Δ), was then defined by subtracting the future from the current invasive species diversity/ evenness estimates. This Δ analysis rescales the output on a -1 to $+1$ scale, where -1 is defined as areas of current, highly suitable, invasive habitat without any future invasive suitability, and $+1$ is defined as areas of the greatest invasive suitability change between the current and future SDM compilations.

A measure of potential habitat degradation, the Additive Invasibility Index (*AII*) was then developed by removing all negative scores from the Δ analysis and adding only areas that have an increase in habitat suitability (as defined by Δ scores >0), to the baseline E. This analysis assumes that actually or potentially invaded/degraded habitat is unlikely to revert to non-degraded habitat. In defining invasibility using a set of species that have evolved in relatively dissimilar habitat (Table 1), we expected the overall invasible area to approximate one.

Following the development of the *AII*, a jackknife test was conducted to assess the degree of information each EMIP species adds to the *AII*. The metric is essentially a measure of area increase per species defined by assessing the significant difference of each location in the EMIPs SDM as compared to the overall *AII* distribution.

f. Defining the Invasibility of Hawaii's Critical Habitat. We overlaid each SDM and invasibility metric with all proposed and designated critical habitat (essentially the compilation of all non-overlapping habitat in [87–102]), to assess the utility of the invasibility metrics as well as to apply these metrics onto areas of conservation concern. Critical habitat is a federal management unit that identifies areas essential for the survival and recovery of 416 endemic Hawaiian threatened and endangered species, including plants, birds and invertebrates. The polygons defined for the species were compiled into a single critical habitat metric because the EMIPs defined here have the potential to significantly alter native habitat, and thus are relevant to all species dependent on that habitat. Vulnerability of individual species/guilds was not defined within this analysis. Only polygons associated with critical habitat, as defined within the extent of the main Hawaii islands (extent shown in Fig. 1), were used to develop the critical habitat metric analyzed here. The overall area (in km^2) of both the baseline and future SDMs/invasibility (and their respective Δ) was assessed for the main Hawaiian islands and critical habitat.

The proportion of habitat defined by the projected invasibility indices, and SDMs, was also defined for the main Hawaiian islands, and within critical habitat. To define the proportional habitat per EMIP SDM/invasibility metric, the thresholded area of each was divided by the overall land area assessed for the State of Hawaii (16,677 km^2) and within Hawaii's critical habitat (3,000 km^2).

g. Google Earth .kmz output. Using the R package 'plotKML' [103] all species models and invisability metrics were output in the .kmz format such that they can be interactively accessed and viewed in Google Earth. Individual species response plots and variable importance boxplots are plotted within each species .kmz to allow for interactive assessments of each SDM. The continuous thresholded SDM projections, and the baseline and future committee averaging metrics are plotted to help understand the projection variance within the projections. A separate .kmz file was also created to interactively present each overall invasibility metric for each species, as well as an average committee averaging depiction defined over all species. An outline of habitats was also overlaid with each SDM/Invasibility matric within each .kmz file to assess invasive suitability structure within each polygon.

D. Caveats and Modeling Limitations

a. Model Projections and Collections. In this analysis climate space is not only determined by the niche preference of each organism, but also by the distribution and extent of the collection regime. This collection regime was mostly determined by organizations that emphasize collection of invasive species data within areas of conservation concern. Although we attempted to account for this collection bias by using similarly collected background points, species location collection constraints may still be biasing the results in a number of ways. First, there were sometimes clear differences between the expert defined [43] and point derived [42] habitat types, especially for *SphCoo* or *PasTar* (Table 1). For these species this discrepancy may be because point locations were within dry climate regions and growing in localities that are anthropogenically modified to favor these species. Second, records for some species were highly unevenly distributed among islands. For example, despite *SphCoo* occurring on all major islands, most records were from Maui, Molokai, and Lanai, while Oahu and Kauai had none. By having records concentrated in the center of the archipelago, most values were in the mid-domain for variables that varied subtly (but consistently) from one end of the

archipelago to the other (particularly Bio2 and Bio4, which relate to temperature seasonality). The result is a set SDMs with high suitability scores on those islands where records occur, and a poor ability to project to other islands. Finally, because establishment dates may vary according to the island in which they have invaded, the individual EMIP analyses may only define a subset of the available habitat due to non-equilibrium on other islands. However, E and AII should account for some of the variance in EMIP collections because the individual SDMs are compiled into a single metric, and the distributions are still broadly applicable to invasibility.

b. Critical Habitat Comparisons. The critical habitat described here has been designated through a formal rulemaking procedure under the Endangered Species Act of 1973 [87–102] and cannot be modified without additional formal rulemaking. Thus, critical habitat does not directly account for projected changes in habitat degradation. Because these designations are semi-permanent, the comparison is valid in a theoretical sense; however, the future interactions are purely hypothetical. We attempt to account for this lack of predictive ability in critical habitat designation by using an overall invasibility metric (the AII) that adds both baseline and future invasibility projections together. This metric predicts habitat degradation and projects invasive incursion. By overlaying the AII with critical habitat we attempt to account for how competitor movement and habitat degradation may affect Hawaii's critical habitat, and thus identify areas that will likely recede due indirectly (i.e. through competitive interaction) to climate change. We are currently working on a more expansive analysis of the impact of climate change on listed plant habitats. This work will help to evaluate the future efficacy of currently designated critical habitat.

Results

All EMIPs selected to define the invasibility index had a mean risk assessment score of 18.8 and establishment time *circa* 1908, (Table 1) indicating that on average they are in the high risk category [19], and likely close to environmental equilibrium [44]. The AUC and TSS evaluation statistics (Fig. 3) were indicative of highly descriptive models (AUC>0.8, TSS>0.5) per modeling approach (GBM, MAXENT, RF). Response plots (Fig. 4) developed per modeling approach, per EMIP, indicated how the response of the developed SDM varied across each environmental covariate. Multiple, EMIP SDMs differed in the manner in which each environmental covariate was used (Fig. 4), but this discrepancy is to be expected given the modeling methods employed [79]. Species-specific responses to each environmental covariate and variable importance boxplots (for all covariates) are further visualized in Files S1–S17.

The niche overlap metric Warrens I is shown in Fig. 5. Most species were found to significantly differ from a distribution developed using a random subset of their compiled points (Fig. 5). Of the 122 multi-species overlap comparisons, only eight were found to be relatively equivalent ($p>0.05$) (see crossed-out comparisons in Fig. 5) and none were found to significantly differ with an overlap value >0.7.

The six different metrics developed to help model invasibility are shown in Fig. 6. The uncorrected H for both baseline and future projections (Fig. 6A–B) shows estimates of the invasive species diversity per site. The ΔE (Fig. 6E) illustrates areas of both increased and decreased EMIP suitability, but this decrease in projected invasive diversity probably does not correlate to a decrease in habitat degradation/invasibility. Figure 6F is a compilation of both the current and future projected invasive diversity indices compiled into a single metric. These metrics defining invasibility, as well as a compiled species committee averaging metric for both baseline and future projections, can be interactively visualized in File S18. The SDMs and both invasibility metrics (E and AII) predict an increase of invasion into Hawaii's upper elevation areas (Fig. 6A–F and Files S1–S18).

The results of the jackknife assessment indicated that four species (*MicCal*, *PsiCat*, *LeuLeu* and *MorFay*) added the greatest amount of novel information (additive area), with *MicCal* accounting for ~30% of the novel additive area in the AII (Fig. 7). While these four species may add the most area to the AII, the addition of all other species adds to the projected intra-area diversity defined within the AII, therefore we did not subset the AII to only account for the density and diversity of these four species.

The actual and proportional area of each EMIP SDM and invasibility metric within Hawaii and Hawaii's critical habitat are defined in Table 3. Table 3 shows areas of the thresholded suitability metrics and, as such, variance of suitability (a correlate of density variance) may vary throughout the defined metric. This is relevant for the AII, where the proportional habitat defined within Hawaii, and within Hawaii's critical habitat, is almost completely overlapping (~0.97 for critical habitat/Hawaii vs. AII). The E metric showed an overall decrease in habitat area for Hawaii and Hawaii's critical habitat (-76.52 km^2 and -16.70 km^2 respectively) indicating that in the future some of the EMIP SDMs may lose some novel suitable habitat. This is true for *MorFay*, *PanMax*, *PenSet* and *SetPal*, and is consistent between the overall defined area in Hawaii and Hawaii's critical habitat (see Δ Area in Table 3). Although there is some loss in overall invaded habitat, this loss proves minor (~1%). The SDM defined for *PasTar* is descriptive of the greatest amount of suitable habitat throughout Hawaii and Hawaii's critical habitat (Table 3), whereas *MicCal*, the organism that described the greatest amount of novel habitat added to the AII, is only the second most widely projected EMIP (Table 3 and Fig. 7).

Discussion

By identifying, analyzing and combining the geographic distribution of a set of highly invasive ecosystem altering species we have developed an invasibility index that describes potential habitat degradation and models invasive distribution/incursion through time. With these indices (E and AII), we have identified potential habitat degradation within management areas essential to the conservation of threatened and endangered species across the Hawaiian archipelago, as well as described the development of a metric that may be useful elsewhere. We have found that although the modeled distribution of the EMIPs may recede or expand by end of century, the area of the AII and E affecting areas designated as critical habitat is similar for both current and future scenarios (Table 3). Although the E and AII indicate that area changes are modest, there are substantial projected differences between the current and future scenarios within critical habitat. The actual landscape area available for occupation by these 17 invasive species increases by ~11% in 2100 (Table 3, AII minus the baseline E), due to climate change. For critical habitat this increase is about 12% (critical habitat AII minus the critical habitat baseline E). In fact, invasibility is predicted to increase in Hawaii's upper elevation areas (Fig. 6A–F and Files S1–S18), a zone where most of Hawaii's native species already have been relegated [39]. It is notable that critical habitat has already been designated for many of these upper elevation ecosystems (see Files S1–S18), so remediation of invasive species within, and at the boundaries of, these habitats will be critical in the coming decades.

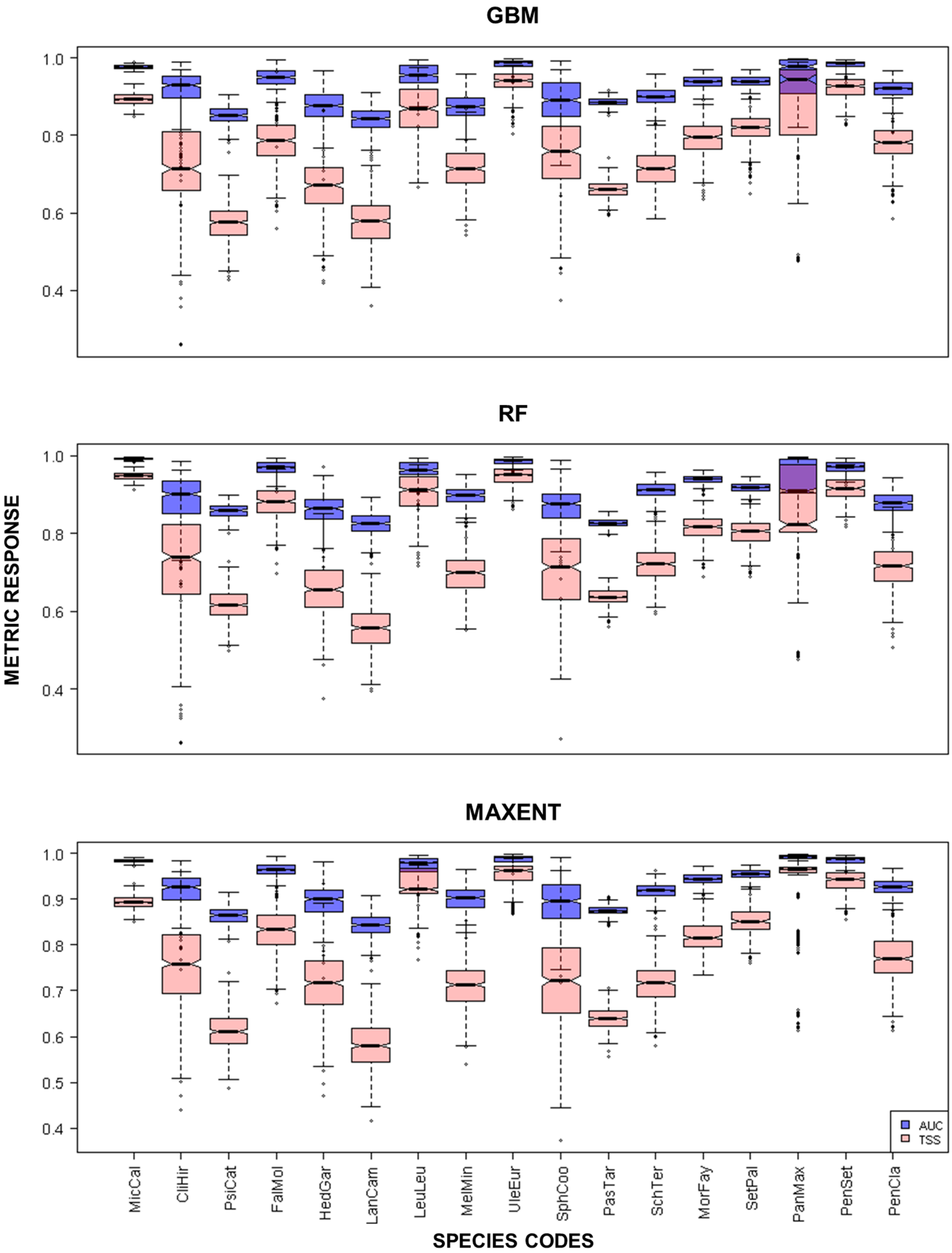

Figure 3. Boxplots representing the variability in each model validation metric (AUC and TSS). Boxplots are shown for each modeling approach used (GBM, Maxent and RF) and compiled for all 500 replicates. The AUC statistic ranges from 0 to 1, where 0.5 characterizes a model no better than that defined for a random distribution of presence points. The TSS validation metric ranges from −1 to 1, where a model with a score of 0 is no better than random. Notches within each boxplot delineate the 95% probability distribution.
doi:10.1371/journal.pone.0095427.g003

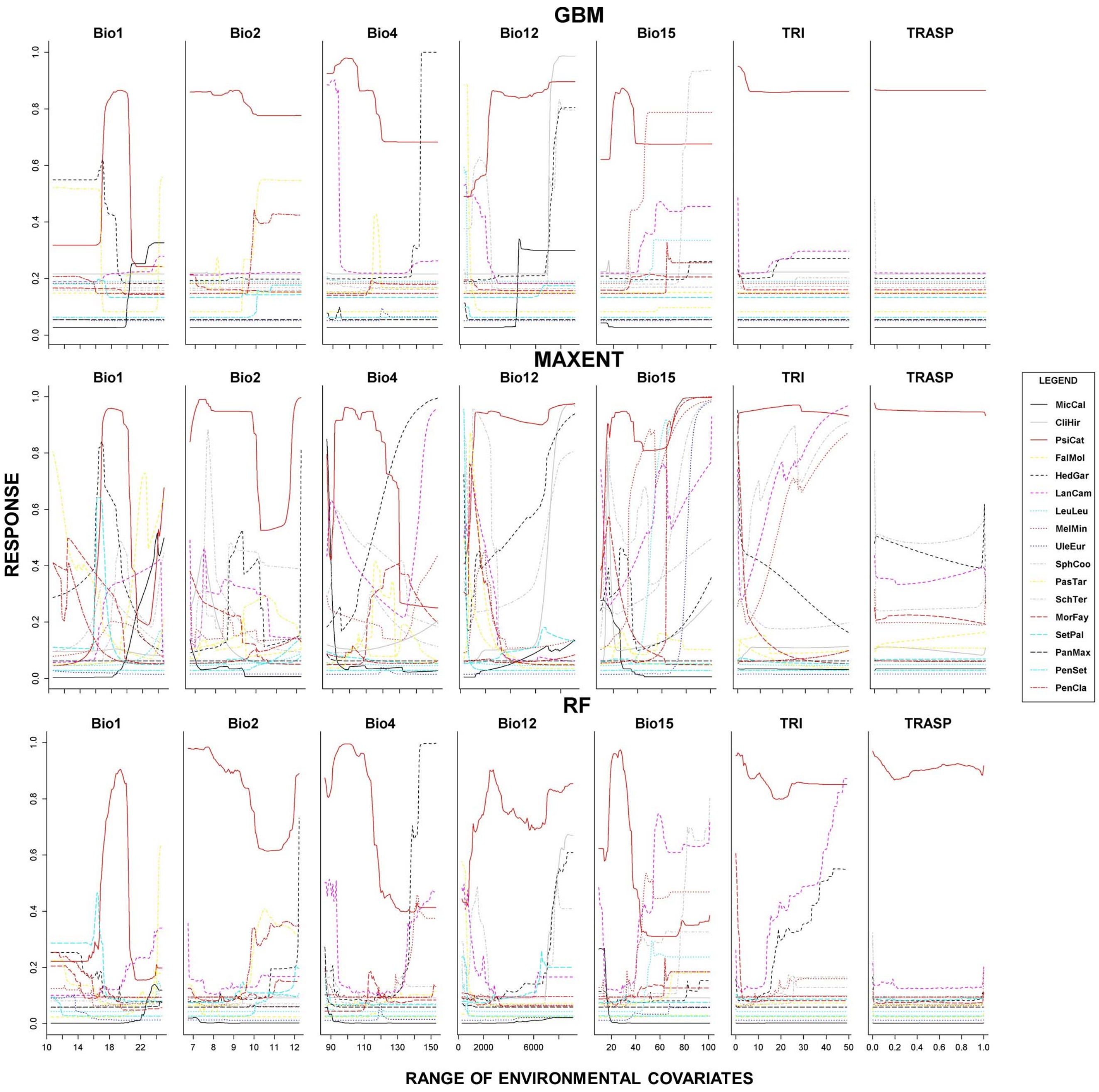

Figure 4. The average response of each species distribution model to each environmental covariate over 500 replicates. The x-axis defines each covariates environmental range, whereas the y-axis delineates the model response.
doi:10.1371/journal.pone.0095427.g004

The invasibility shift modeled here is consistent with other work characterizing the migration of native and invasive species in Hawaii and elsewhere [17,84,104–107]. However, our results show that latitudinal migration to maintain climatic equilibria under climate change, such as that found in North America [108,109] and Australia, [32,33,48], will likely not affect Hawaii's native or invasive species given its limited latitudinal variation. In Hawaii, under increasing temperatures, plant species need to migrate to upper elevation habitat to find temperature equivalent zones that encompass increasingly smaller areas [7].

Although the area most species occupied increased in size when projected into the future (Table 3), four species decreased in area within both Hawaii and the areas within Hawaii designated as critical habitat. Of these, two highly invasive plants occupying upper elevation wet forest habitats [110–113], *MorFay* and *SetPal*, had the most drastic decreases in suitable acreage between current and future projections. These organisms seem to occupy the limits of the wet forest climatic regime, therefore, as similar climate space migrates to upper elevations the projected range of the organisms will likely contract, much like that of native species [114]. These results seem to contradict that of Yelenick and D'Antonio [111], where it was shown that the nitrogen fixing *MorFay* more easily invades low nitrogen, invasive grass (*MelMin*) dominated habitat, and does so more readily than *Acacia koa* (Fabaceae). Their work

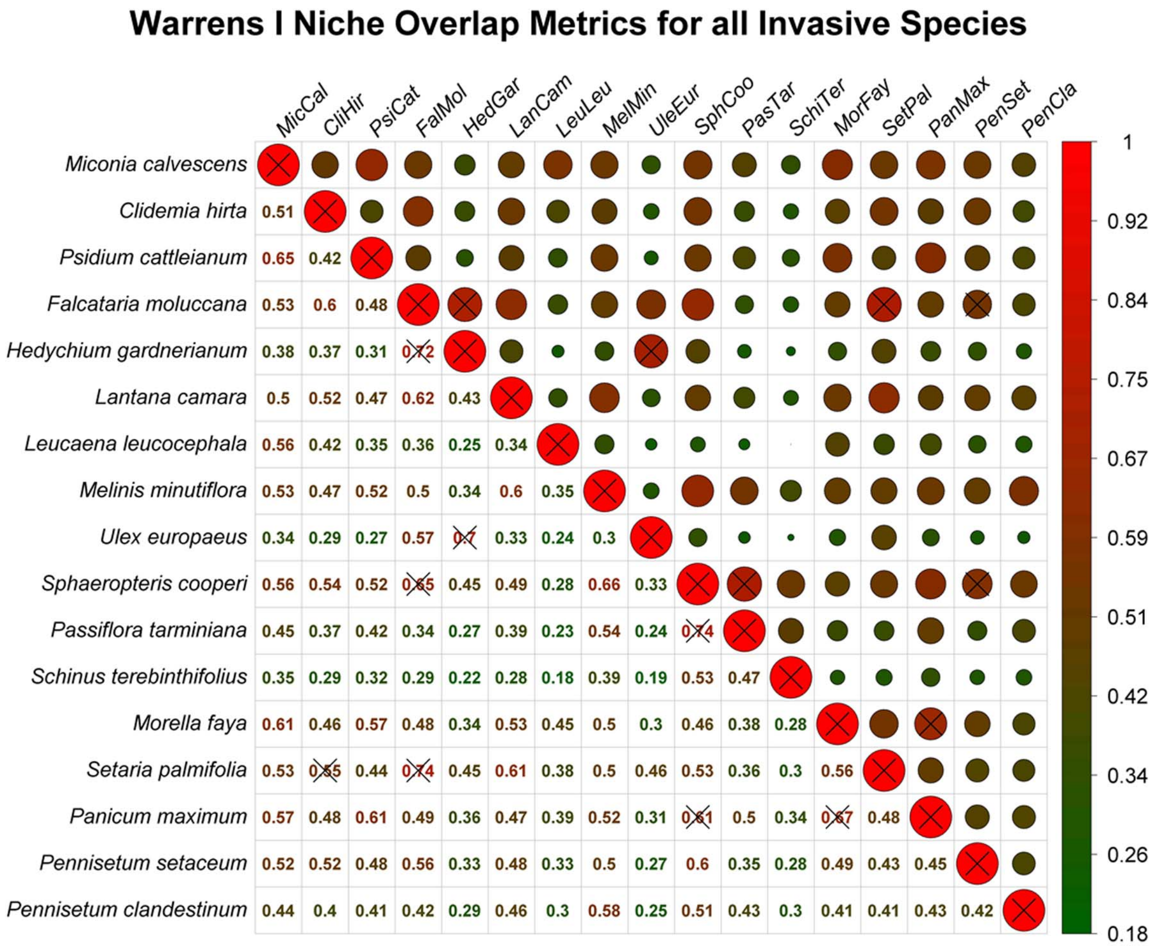

Figure 5. Warrens *I* niche overlap metric per EMIP. The upper diagonal shows the variation in niche overlap using circle size and color (low overlap is shaded green; high overlap is shaded red; extent of overlap is indicated by dot size and red or green color saturation). The lower diagonal gives the actual overlap metric as colored by the scaling graphic. EMIP species names are here represented by both the complete scientific Latin name (y-axis) and its coded counterpart (upper x-axis) as defined in Table 1. All insignificant (p>0.05) niche overlap metrics, as derived from a niche equivalency test, are indicated with an "X".
doi:10.1371/journal.pone.0095427.g005

contends that *MorFay* will expand its distribution in the absence of competition with *MelMin* and climate change [111]. Although seemingly contradictory, their scenario is still likely given that, by design, the models defined here are projections of invasive habitat suitability and thus project habitat outside of currently invaded areas. So, although future climatic suitability of habitat may contract, expansion may continue as resource competition (e.g. between *MelMin* and *MorFay*) [111] becomes greater.

In the Endangered Species Act of 1973 critical habitat is defined as a geographic area that has physical and biological characteristics needed to support viable populations of the species and that is considered to be essential to the survival of the species for which it was designated. Designated critical habitat receives protection from Federal actions that would adversely modify it. Divergence of these biotic and administrative features of critical habitat occurs once biotic and abiotic threats modify these ecosystems to a point where the critical habitat can no longer support viable populations of the endangered or threatened species for which it was designated. Because these formal habitat designations do not account for projected changes in habitat degradation there are limitations to their use in recovering endangered or threatened species [115], especially in regards to whether in the future that habitat is still biologically relevant to the organism(s) that designation was meant to protect. Critical habitat designations that have not taken climate change effects into account will need to be reassessed and possibly revised as climate change progresses; a complex and time consuming process [116–118]. New designations of critical habitat that incorporate climate change impacts into the designation process will enhance the usefulness of critical habitat under future climate change conditions and will minimize future resource expenditures on this administrative process. The metrics developed here can aid in evaluating the current and future value of critical habitat, and in directing resources to managing those invasive species that will result in the greatest protection of habitat that is critical to the long term survival and recovery of endangered and threatened species. As outlined by Peters and Darling [115] evaluating the potential for habitat degradation in any type of native sanctuary is critical to successfully adapting management strategies to climate change.

Understanding projected habitat changes (through climate change or invasive incursion) is especially pertinent to native dominated Wet and Mesic zones on Maui and Hawaii islands, where high elevation areas above current critical habitat may serve as future critical habitat under continued warming. However, this upslope shift will likely be determined by changes to the height and frequency of the trade wind inversion [105,119] that caps cloud

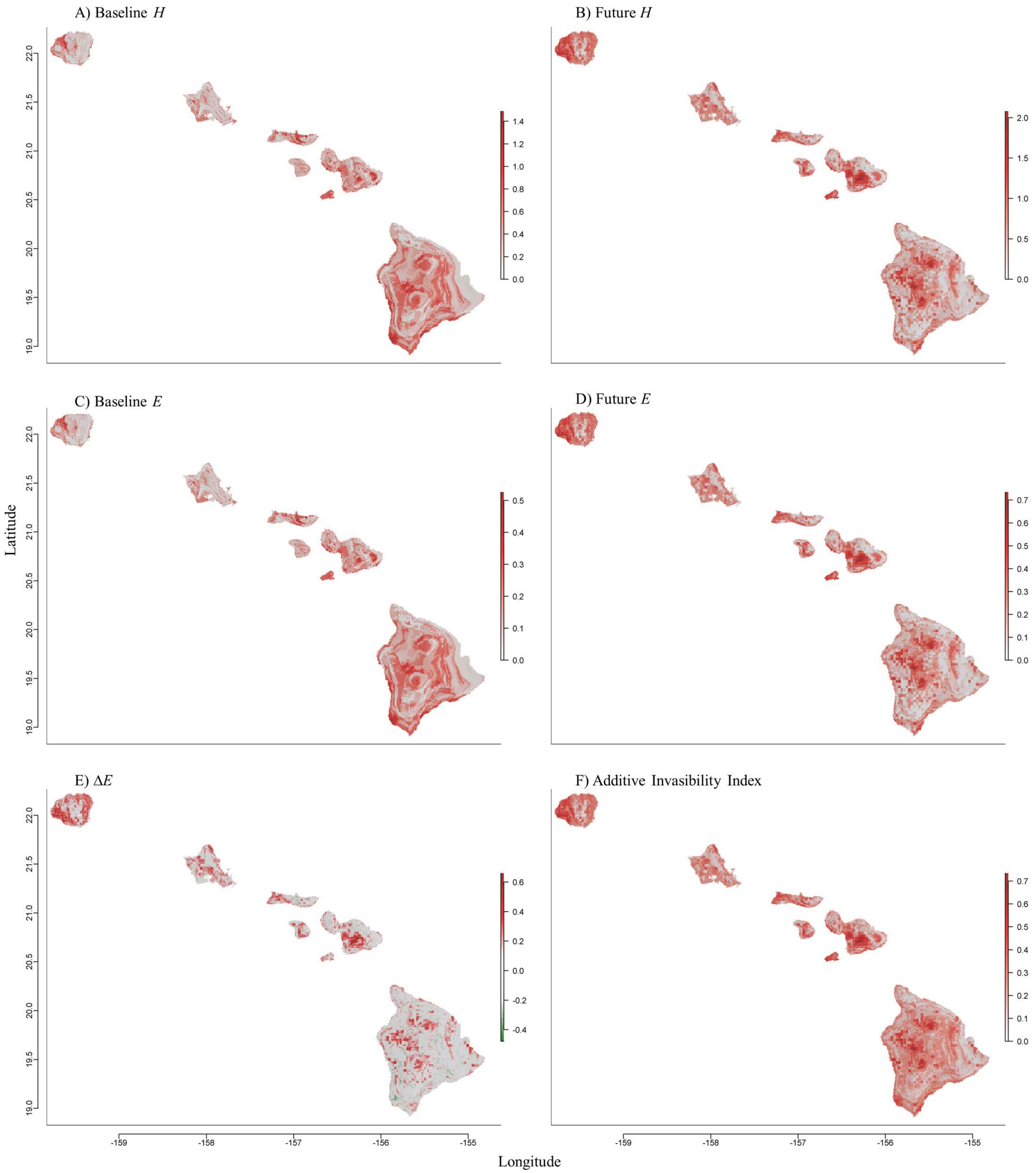

Figure 6. Baseline and Future invasibility metrics defining the invasive species diversity per site. The projected current and future diversity (*H*) and species evenness metrics (*E*) (A–D) are shown. The change (Δ) in evenness (Δ*E* = Future *E* – Baseline *E*), shows areas of both increased (red) and decreased (green) projected invasibility over time (E). The Additive Invasibility Index (*AII*) (F) is the compiled Baseline and Future *E*. Both single species models and compiled graphic (Invasibility) outputs can be interactively viewed in Files S1, S2, S3, S4, S5, S6, S7, S8, S9, S10, S11, S12, S13, S14, S15, S16, S17, S18.
doi:10.1371/journal.pone.0095427.g006

heights typically below 2000 meters and leads to an abrupt shift from wet/mesic habitats to dry/arid mountain tops. Whether the habitat recedes or migrates, resource competition with invasive species will likely persist at the habitats' periphery and potentially spread to the interior if facilitated by biotic or abiotic factors. This will be especially apparent in dry forests; a habitat type regulated

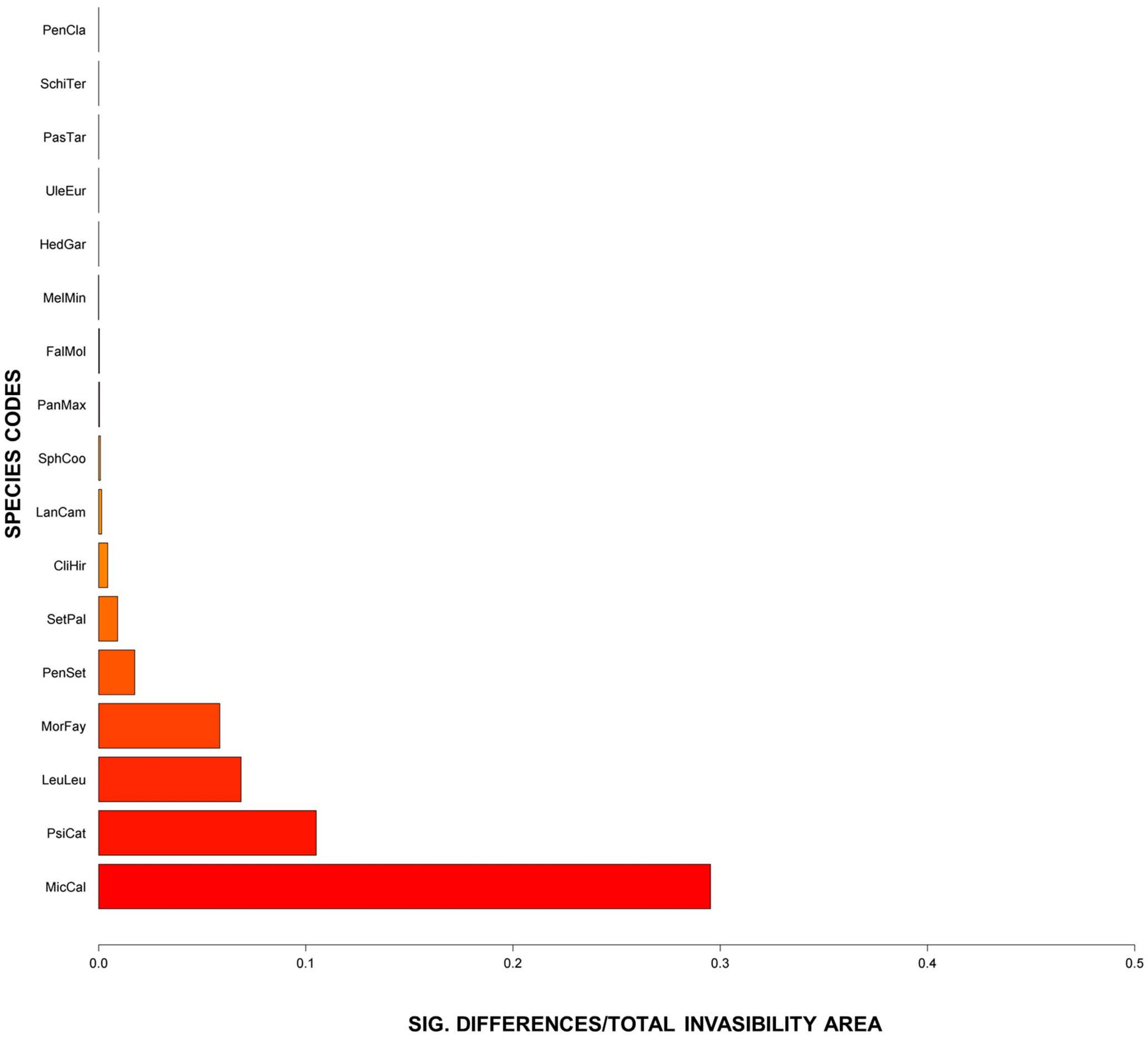

Figure 7. Jackknife validation of the EMIPs significance as compared to the invasibility metric. The y-axis defines, on a 0 to 1 scale, the number of significantly different pixels as compared to the total invasibility area metric. This metric estimates the proportion of novel habitat defined per species.
doi:10.1371/journal.pone.0095427.g007

more by anthropogenic mediated interactions (habitat destruction/fragmentation, invasion, and degradation) than by climate [120]. As such, the invasibility index developed here may help in evaluating habitats that migrate to climatically equivalent areas, but will still face continued environmental stress from invasive competition and incursion.

Given the focus of collecting species location data within areas of high conservation value, our results bear the greatest relevance to similar areas across the archipelago. As such, we recommend that end users constrain the metrics by areas of high conservation value. Although in this research we have focused on the overlap of invasibility with critical habitat, there are other areas of conservation concern such as federal and state refuge/park land, national forests and private land conservancies, all of which have easily accessible layers in Google Earth that can be overlaid with the metrics defined here. The addition of these interactive layers increases the utility of these invasive SDM metrics to conservation managers by helping to spatially refine invasive species management.

A broad-scale systematic sampling effort for all species would ultimately improve the individual EMIP SDM predictions and projections [72,121]. Although the projections would improve with increased survey effort, the resulting models may still underrepresent future distributions due to (as yet undocumented) EMIP tolerance to non-analog climate space. By developing an iterative modeling approach within the R statistical environment we have created a toolset that will allow us to re-project the SDMs with increasing accuracy as more data becomes available from surveys and analysis of species climate tolerance. So, while we have analyzed a single future climate scenario, our models can be expanded to include multiple emission scenarios and different time steps to explore a range of possible outcomes. With the future

Table 3. The actual and proportional area of each thresholded species distribution model and projected Invasibility metric within Hawaii and in Hawaii's critical habitat.

		Overall Area (km^2)			*Area in Critical Habitat (km^2)*[4]			*Prop. To HI*[2,3]		*Prop. To CH*[2,3]	
	**Baseline (B)**	**Future (2100) (F)**	**Future Area Rank**	**Δ (F-B)**[1]	**Baseline (BC)**	**Future (2100) (FC)**	**Δ (FC-BC)**[1]	**Baseline**	**Future (2100)**	**Baseline**	**Future (2100)**
**PasTar**	5114.63	6309.72	1	1195.09	649.81	670.65	20.84	0.31	0.38	0.22	0.22
**HedGar**	509.41	3542.27	2	3032.86	292.07	709.04	416.97	0.03	0.21	0.10	0.24
**LanCam**	2010.76	3003.88	3	993.11	340.07	723.31	383.24	0.12	0.18	0.11	0.24
**PsiCat**	1901.38	2454.60	4	553.23	483.65	646.56	162.90	0.11	0.15	0.16	0.22
**PenCla**	2063.50	2391.00	5	327.50	339.40	440.96	101.55	0.12	0.14	0.11	0.15
**MicCal**	2084.09	2137.24	6	53.16	317.67	425.02	107.35	0.12	0.13	0.11	0.14
PenSet[5]	1954.18	1953.67	***7***	***−0.52***	214.23	151.51	***−62.72***	0.12	0.12	0.07	0.05
**MelMin**	1049.23	1737.33	8	688.10	313.48	441.14	127.66	0.06	0.10	0.10	0.15
**SphCoo**	425.97	1241.47	9	815.50	53.27	277.50	224.22	0.03	0.07	0.02	0.09
**SchiTer**	959.42	1176.02	10	216.60	196.71	213.48	16.77	0.06	0.07	0.07	0.07
**LeuLeu**	727.21	1046.33	11	319.12	55.72	118.00	62.28	0.04	0.06	0.02	0.04
**FalMol**	470.24	804.67	12	334.43	51.25	101.11	49.86	0.03	0.05	0.02	0.03
**UleEur**	164.40	724.52	15	560.13	106.31	260.28	153.97	0.01	0.04	0.04	0.09
**CliHir**	189.85	515.16	14	325.31	112.39	187.75	75.36	0.01	0.03	0.04	0.06
PanMax[5]	263.71	248.04	***15***	***−15.67***	22.74	15.71	***−7.03***	0.02	0.01	0.01	0.01
MorFay[5]	402.49	23.64	***16***	***−378.85***	139.33	4.34	***−134.99***	0.02	0.00	0.05	0.00
SetPal[5]	297.74	0.26	***17***	***−297.48***	152.19	0.26	***−151.93***	0.02	0.00	0.05	0.00
**Shannon's Evenness (*E*)**	14301.61	14225.09	-	−76.52	2562.49	2545.79	−16.70	0.86	0.85	0.85	0.85
**Additive Invasibility Index (*AII*)**	16138.70				2910.76			0.97		0.97	

[1]The change (Δ) in area from baseline to future projections.
[2]Defines the proportion of suitable habitat falling within Hawaii and Hawaii's critical habitat.
[3]Area of Hawaii calculated as 16,677 km^2.
[4]Critical Habitat areas calculated to be 3,000 km^2.
[5]Habitat Modifying Invasive Plants (EMIP's) bolded and italicized indicated a reduction in habitat between the baseline and future scenarios.
doi:10.1371/journal.pone.0095427.t003

releases of such climatic datasets we plan to update our analyses accordingly. The code used to define the models and model outputs can be found in the supplementary materials (Code S1).

In summary, we have developed a reproducible methodology to identify species and areas of conservation concern in Hawaii, a region characterized by both high endemic biodiversity and invasive pressure. The resulting models are novel in that they are the first ensemble of multiple EMIPs to geographically delineate, and rigorously quantify, invasibility in Hawaii. Given Hawaii's extraordinarily high endemic biodiversity [39] and quantity of endangered species [34,35], delineating and projecting areas of increased invasive pressure on native resources is of paramount importance.

Supporting Information

Code S1 R code used for the analyses presented in this manuscript.
(ZIP)

File S1 Baseline and Future Species Distribution Ensemble Model (SDM EM) and associated validation metrics for *Clidemia hirta* as depicted in a Google Earth .kmz file.
(KMZ)

File S2 Baseline and Future Species Distribution Ensemble Model (SDM EM) and associated validation metrics for *Falcataria moluccana* as depicted in a Google Earth .kmz file.
(KMZ)

File S3 Baseline and Future Species Distribution Ensemble Model (SDM EM) and associated validation metrics for *Hedychium gardnerianum* as depicted in a Google Earth .kmz file.
(KMZ)

File S4 Baseline and Future Species Distribution Ensemble Model (SDM EM) and associated validation metrics for *Lantana camara* as depicted in a Google Earth .kmz file.
(KMZ)

File S5 Baseline and Future Species Distribution Ensemble Model (SDM EM) and associated validation metrics for *Leucaena leucocephala* as depicted in a Google Earth .kmz file.
(KMZ)

File S6 Baseline and Future Species Distribution Ensemble Model (SDM EM) and associated validation metrics for *Melinis minutiflora* as depicted in a Google Earth .kmz file.
(KMZ)

File S7 Baseline and Future Species Distribution Ensemble Model (SDM EM) and associated validation metrics for *Miconia calvescens* as depicted in a Google Earth .kmz file.
(KMZ)

File S8 Baseline and Future Species Distribution Ensemble Model (SDM EM) and associated validation metrics for *Morella faya* as depicted in a Google Earth .kmz file.
(KMZ)

File S9 Baseline and Future Species Distribution Ensemble Model (SDM EM) and associated validation metrics for *Panicum maximum* as depicted in a Google Earth .kmz file.
(KMZ)

File S10 Baseline and Future Species Distribution Ensemble Model (SDM EM) and associated validation metrics for *Passiflora tarminiana* as depicted in a Google Earth .kmz file.
(KMZ)

File S11 Baseline and Future Species Distribution Ensemble Model (SDM EM) and associated validation metrics for *Pennisetum clandestinum* as depicted in a Google Earth .kmz file.
(KMZ)

File S12 Baseline and Future Species Distribution Ensemble Model (SDM EM) and associated validation metrics for *Pennisetum setaceum* as depicted in a Google Earth .kmz file.
(KMZ)

File S13 Baseline and Future Species Distribution Ensemble Model (SDM EM) and associated validation metrics for *Psidium cattleianum* as depicted in a Google Earth .kmz file.
(KMZ)

File S14 Baseline and Future Species Distribution Ensemble Model (SDM EM) and associated validation metrics for *Schinus terebinthifolius* as depicted in a Google Earth .kmz file.
(KMZ)

File S15 Baseline and Future Species Distribution Ensemble Model (SDM EM) and associated validation metrics for *Setaria palmifolia* as depicted in a Google Earth .kmz file.
(KMZ)

File S16 Baseline and Future Species Distribution Ensemble Model (SDM EM) and associated validation metrics for *Sphaeropteris cooperi* as depicted in a Google Earth .kmz file.
(KMZ)

File S17 Baseline and Future Species Distribution Ensemble Model (SDM EM) and associated validation metrics for *Ulex europaeus* as depicted in a Google Earth .kmz file.
(KMZ)

File S18 Baseline and Future invasibility indices (*AII*, *H*, and *E*) and associated validation metrics for all invasive species modeled in this study, as depicted in a Google Earth .kmz file.
(KMZ)

Acknowledgments

The findings and conclusions of this article are those of the authors and do not necessarily represent the views of the U.S. Fish and Wildlife Service. We would like to acknowledge L. Mehrhoff (Pacific Island Fish and Wildlife Office) and J. Burgett (Pacific Island Climate Change Cooperative) for their insightful conversations and support of the work; D. Georges for his help with the R package Biomod2; and Forest and Kim Star, D. Clarke, V. Caraway and J. Fisher for allowing us to use their photographs of invasive species in Hawaii. The authors would also like to thank The Nature Conservancy, Hawaii Volcanoes National Park, the Hawaiian Islands Invasive Species Councils (KISC, OISC, MISC, and HISC), the U.S. Geological Survey and the U.S. FWS National Wildlife Refuges for providing data and recommendations concerning the manuscript. We are grateful for the support of the U.S. FWS Ecological Services, the USGS Pacific Island Ecosystems Research Center, the Pacific Islands Climate Change Cooperative and all of the other author organizations that volunteered significant effort and resources to make this project possible. Finally, we would like to sincerely thank P. Adam and two anonymous reviewers for their excellent recommendations and guidance. Any use of trade, firm, or product names is for descriptive purposes only and does not imply endorsement by the U.S. Government.

Author Contributions

Conceived and designed the experiments: LBF AEV FAA SEM. Analyzed the data: AEV FAA LBF. Contributed reagents/materials/analysis tools: SEM JDJ SOG GAK JPP. Wrote the paper: AEV FAA LBF.

References

1. Mascaro J, Hughes RF, Schnitzer SA (2012) Novel forests maintain ecosystem processes after the decline of native tree species. Ecol Monogr 82: 221–228.
2. Mack RN, Simberloff D, Mark Lonsdale W, Evans H, Clout M, et al. (2000) Biotic invasions: causes, epidemiology, global consequences, and control. Ecol Appl 10: 689–710.
3. Asner GP, Vitousek PM (2005) Remote analysis of biological invasion and biogeochemical change. Proc Natl Acad Sci 102: 4383–4386.
4. Funk JL (2005) Hedychium gardnerianum Invasion into Hawaiian Montane Rainforest: Interactions Among Litter Quality, Decomposition Rate, and Soil Nitrogen Availability. Biogeochemistry 76: 441–451. doi:10.1007/s10533-005-7657-7
5. Vitousek PM, Walker LR, Whiteaker LD, Mueller-Dombois D, Matson PA (1987) Biological invasion by Myrica faya alters ecosystem development in Hawaii. Science 238: 802–804.
6. Asner GP, Hughes RF, Vitousek PM, Knapp DE, Kennedy-Bowdoin T, et al. (2008) Invasive plants transform the three-dimensional structure of rain forests. Proc Natl Acad Sci 105: 4519–4523.
7. Penuelas J, Sardans J, Llusià J, Owen SM, Carnicer J, et al. (2009) Faster returns on "leaf economics" and different biogeochemical niche in invasive compared with native plant species. Glob Change Biol 16: 2171–2185. doi:10.1111/j.1365-2486.2009.02054.x
8. Vitousek PM, Tweiten MA, Kellner J, Hotchkiss SC, Chadwick OA, et al. (2010) Top-Down Analysis of Forest Structure and Biogeochemistry Across Hawaiian Landscapes. Pac Sci 64: 359–366. doi:10.2984/64.3.359
9. Hughes RF, Denslow JS (2005) Invasion by a N2-fixing tree alters function and structure in wet lowland forests of Hawaii. Ecol Appl 15: 1615–1628.
10. Tweiten MA, Hotchkiss SC, Vitousek PM, Kellner JR, Chadwick OA, et al. (2013) Resilience against exotic species invasion in a tropical montane forest. J Veg Sci: early view. doi:10.1111/jvs.12112
11. Mealor BA, Hild AL (2007) Post-invasion evolution of native plant populations: a test of biological resilience. Oikos 116: 1493–1500. doi:10.1111/j.0030-1299.2007.15781.x
12. Willis CG, Ruhfel BR, Primack RB, Miller-Rushing AJ, Losos JB, et al. (2010) Favorable Climate Change Response Explains Non-Native Species' Success in Thoreau's Woods. PLoS ONE 5: e8878. doi:10.1371/journal.pone.0008878
13. Ricciardi A, Jones LA, Kestrup ÅM, Ward JM (2011) Expanding the propagule pressure concept to understand the impact of biological invasions. Fifty Years Invasion Ecol Leg Charles Elton: 225–235.
14. Huang D, Haack RA, Zhang R (2011) Does Global Warming Increase Establishment Rates of Invasive Alien Species? A Centurial Time Series Analysis. PLoS ONE 6: e24733. doi:10.1371/journal.pone.0024733
15. Ziska LH, Blumenthal DM, Runion GB, Hunt ER, Diaz-Soltero H (2010) Invasive species and climate change: an agronomic perspective. Clim Change 105: 13–42. doi:10.1007/s10584-010-9879-5
16. Dukes JS (2011) Responses of invasive species to a changing climate and atmosphere. Fifty Years Invasion Ecol Leg Charles Elton Blackwell Oxf: 345–357.
17. Benning TL, LaPointe D, Atkinson CT, Vitousek PM (2002) Interactions of climate change with biological invasions and land use in the Hawaiian Islands: modeling the fate of endemic birds using a geographic information system. Proc Natl Acad Sci 99: 14246–14249.
18. Levine JM (2000) Species Diversity and Biological Invasions: Relating Local Process to Community Pattern. Science 288: 852–854. doi:10.1126/science.288.5467.852
19. Daehler CC, Denslow JS, Ansari S, Kuo H-C (2004) A Risk-Assessment System for Screening Out Invasive Pest Plants from Hawaii and Other Pacific Islands. Conserv Biol 18: 360–368.
20. Stohlgren TJ, Binkley D, Chong GW, Kalkhan MA, Schell LD, et al. (1999) Exotic plant species invade hot spots of native plant diversity. Ecol Monogr 69: 25–46.
21. Boelman NT, Asner GP, Hart PJ, Martin RE (2007) Multi-trophic invasion resistance in Hawaii: Bioacoustics, field surveys, and airborne remote sensing. Ecol Appl 17: 2137–2144.
22. Zenni RD, Nuñez MA (2013) The elephant in the room: the role of failed invasions in understanding invasion biology. Oikos: no–no. doi:10.1111/j.1600-0706.2012.00254.x
23. Fridley J (2011) Invasibility of Communities and Ecosytems. In: Simberloff FD, Rejmánek M, editors. Encyclopedia of Biological Invasions. Berkeley and Los Angelas: University of California Press. pp. 356–360.
24. Rejmánek M (1989) Invasibility of Plant Communities. In: Drake JA, Mooney HA, Castri F di, Groves RH, Kruger FJ, et al., editors. Biological invasions: a global perspective. Chichester, UK.: John Wiley and Sons. pp. 369–388.
25. Bisrat SA, White MA, Beard KH, Richard Cutler D (2012) Predicting the distribution potential of an invasive frog using remotely sensed data in Hawaii. Divers Distrib 18: 648–660. doi:10.1111/j.1472-4642.2011.00867.x
26. Stohlgren TJ, Ma P, Kumar S, Rocca M, Morisette JT, et al. (2010) Ensemble habitat mapping of invasive plant species. Risk Anal 30: 224–235.
27. Trotta-Moreu N, Lobo JM (2010) Deriving the Species Richness Distribution of Geotrupinae (Coleoptera: Scarabaeoidea) in Mexico From the Overlap of Individual Model Predictions. Environ Entomol 39: 42–49. doi:10.1603/EN08179
28. Pineda E, Lobo JM (2009) Assessing the accuracy of species distribution models to predict amphibian species richness patterns. J Anim Ecol 78: 182–190. doi:10.1111/j.1365-2656.2008.01471.x
29. Guisan A, Theurillat J-P (2000) Equilibrium modeling of alpine plant distribution: how far can we go? In: Deil U, Loidi J, editors. Vegetation and climate. A selection of contributions presented at the 42nd Symposium of the International Association of Vegetation Science, Bilbao, Spain, 26–30 July 1999. Gebrüder Borntraeger Verlagsbuchhandlung, Vol. 30. pp. 353–384.
30. O'Donnell J, Gallagher RV, Wilson PD, Downey PO, Hughes L, et al. (2012) Invasion hotspots for non-native plants in Australia under current and future climates. Glob Change Biol 18: 617–629. doi:10.1111/j.1365-2486.2011.02537.x
31. Catford JA, Vesk PA, White MD, Wintle BA (2011) Hotspots of plant invasion predicted by propagule pressure and ecosystem characteristics. Divers Distrib 17: 1099–1110. doi:10.1111/j.1472-4642.2011.00794.x
32. Gallagher RV, Hughes L, Leishman MR, Wilson PD (2010) Predicted impact of exotic vines on an endangered ecological community under future climate change. Biol Invasions 12: 4049–4063. doi:10.1007/s10530-010-9814-8
33. Duursma DE, Gallagher RV, Roger E, Hughes L, Downey PO, et al. (2013) Next-Generation Invaders? Hotspots for Naturalised Sleeper Weeds in Australia under Future Climates. PLoS ONE 8: e84222. doi:10.1371/journal.pone.0084222
34. Pimentel D, Zuniga R, Morrison D (2005) Update on the environmental and economic costs associated with alien-invasive species in the United States. Ecol Econ 52: 273–288. doi:10.1016/j.ecolecon.2004.10.002
35. U.S. National Archives and Records Administration. (2014) e-CFR: Title 50: Wildlife and Fisheries Part 17-Endangered and Threatend Wildlife and Plants Subpart B—Lists. Electron Code Fed Regul. Available: http://www.ecfr.gov/cgi-bin/text-idx?c = ecfr&sid = 186cb0f38a1b1b6770e432a7eba20553&rgn = div8&view = text&node = 50:2.0.1.1.1.2.1.2&idno = 50. Accessed 2014 April 15.
36. Vitousek PM, D'Antonio CM, Loope LL, Rejmánek M, Westbrooks R (1997) Introduced species: A significant component of human-caused global change. N Z J Ecol 21: 1–16.
37. Levine JM, Vilà M, Antonio CMD, Dukes JS, Grigulis K, et al. (2003) Mechanisms underlying the impacts of exotic plant invasions. Proc R Soc Lond B Biol Sci 270: 775–781. doi:10.1098/rspb.2003.2327
38. Gurevitch J, Padilla DK (2004) Are invasive species a major cause of extinctions? Trends Ecol Evol 19: 470–474. doi:10.1016/j.tree.2004.07.005
39. Ziegler AC (2002) Hawaiian natural history, ecology, and evolution. Honolulu, Hawaii: University of Hawaii Press. 476 p.
40. Simberloff D (2013) Invasive species, what everyone needs to know. New York, NY: Oxford University Press. 352 p.
41. Pheloung PC, Williams PA, Halloy SR (1999) A weed risk assessment model for use as a biosecurity tool evaluating plant introductions. J Environ Manage 57: 239–251. doi:10.1006/jema.1999.0297
42. Price JP, Jacobi JD, Gon SM, Matsuwaki D, Mehrhoff L, et al. (2012) Mapping plant species ranges in the Hawaiian Islands-Developing a methodology and associated GIS layers. Open File Report -2012-1192. Honolulu, Hawaii: United States Geological Survey. Available: http://pubs.usgs.gov/of/2012/1192/. Accessed 2014 April 15.
43. Wagner W, Herbst DR, Sohmer S (1999) Manual of the flowering plants of Hawai'i. Honolulu, HI: University of Hawai'i Press: Bishop Museum Press. 1853 p.
44. Vaclavík T, Meentemeyer RK (2012) Equilibrium or not? Modelling potential distribution of invasive species in different stages of invasion. Divers Distrib 18: 73–83. doi:10.1111/j.1472-4642.2011.00854.x
45. Lozier JD, Mills NJ (2011) Predicting the potential invasive range of light brown apple moth (Epiphyas postvittana) using biologically informed and correlative species distribution models. Biol Invasions 13: 2409–2421. doi:10.1007/s10530-011-0052-5
46. Broennimann O, Treier UA, Müller-Schärer H, Thuiller W, Peterson AT, et al. (2007) Evidence of climatic niche shift during biological invasion. Ecol Lett 10: 701–709. doi:10.1111/j.1461-0248.2007.01060.x
47. Beaumont LJ, Gallagher RV, Thuiller W, Downey PO, Leishman MR, et al. (2009) Different climatic envelopes among invasive populations may lead to underestimations of current and future biological invasions. Divers Distrib 15: 409–420. doi:10.1111/j.1472-4642.2008.00547.x
48. Gallagher RV, Beaumont LJ, Hughes L, Leishman MR (2010) Evidence for climatic niche and biome shifts between native and novel ranges in plant species introduced to Australia. J Ecol 98: 790–799. doi:10.1111/j.1365-2745.2010.01677.x
49. Giambelluca TW, Chen Q, Frazier AG, Price JP, Chen Y-L, et al. (2013) Online Rainfall Atlas of Hawai'i. Bull Am Meteorol Soc 94: 313–316. doi:10.1175/BAMS-D-11-00228.1
50. Daly C, Conklin DR, Unsworth MH (2009) Local atmospheric decoupling in complex topography alters climate change impacts. Int J Climatol 30: 1857–1864. doi:10.1002/joc.2007
51. PRISM Climate Group. Oregon State University. Available: http://prism.oregonstate.edu. Accessed 2014 April 15.
52. Team RC (2013) R: A Language and Environment for Statistical Computing. Vienna, Austria. Available: http://www.R-project.org. Accessed 2014 April 15.

53. Hijmans R, Phillips S, Leathwick J, Elith J (2010) Dismo: Species distribution modeling. R Package Version 05-4 Available Internet HttpCRAN R-Proj Orgpackage Dismo.
54. Maune DF, Dewberry C (2010) Digital Elevation Model (DEM) Whitepaper NRCS High Resolution Elevation Data.
55. Hijmans RJ, van Etten J (2010) raster: Geographic analysis and modeling with raster data. R Package Version 1: r948.
56. Evans JS, Cushman SA (2009) Gradient modeling of conifer species using random forests. Landsc Ecol 24: 673–683. doi:10.1007/s10980-009-9341-0
57. Jones KH (1998) A comparison of algorithms used to compute hill slope as a property of the DEM. Comput Geosci 24: 315–323.
58. Wilson MFJ, O'Connell B, Brown C, Guinan JC, Grehan AJ (2007) Multiscale Terrain Analysis of Multibeam Bathymetry Data for Habitat Mapping on the Continental Slope. Mar Geod 30: 3–35. doi:10.1080/01490410701295962
59. Wei T (2013) corrplot: visualization of a correlation matrix. R package version 0.60.
60. Zhang C, Wang Y, Lauer A, Hamilton K (2012) Configuration and Evaluation of the WRF Model for the Study of Hawaiian Regional Climate. Mon Weather Rev 140: 3259–3277. doi:10.1175/MWR-D-11-00260.1
61. Nakicenovic N, Alcamo J, Davis G, de Vries B, Fenhann J, et al. (2000) Special report on emissions scenarios: a special report of Working Group III of the Intergovernmental Panel on Climate Change. Pacific Northwest National Laboratory, Richland, WA (US), Environmental Molecular Sciences Laboratory (US).
62. Phillips SJ, Anderson RP, Schapire RE (2006) Maximum entropy modeling of species geographic distributions. Ecol Model 190: 231–259. doi:10.1016/j.ecolmodel.2005.03.026
63. Breiman L (2001) Random forests. Mach Learn 45: 5–32.
64. Friedman JH (2001) Greedy function approximation: a gradient boosting machine. Ann Stat 29: 1189–1232.
65. Caruana R, Niculescu-Mizil A (2006) An empirical comparison of supervised learning algorithms. In: Cohen WW, Moore A, editors. Proceedings of the 23rd International Conference on Machine learning. Pittsburgh, Pennsylvania: Association for Computing Machinery. pp. 161–168. Available: http://dl.acm.org/citation.cfm?id = 1143865. Accessed 2014 April 15.
66. Cutler DR, Edwards Jr TC, Beard KH, Cutler A, Hess KT, et al. (2007) Random forests for classification in ecology. Ecology 88: 2783–2792.
67. Ortega-Huerta MA, Peterson AT (2008) Modeling ecological niches and predicting geographic distributions: a test of six presence-only methods. Rev Mex Biodivers 79: 205–216.
68. Hastie T, Tibshirani R, Friedman JH (2011) The elements of statistical learning. 2nd ed. New York: Springer. 758 p.
69. Elith J, Phillips SJ, Hastie T, Dudík M, Chee YE, et al. (2011) A statistical explanation of MaxEnt for ecologists. Divers Distrib 17: 43–57. doi:10.1111/j.1472-4642.2010.00725.x
70. Elith J, Leathwick JR, Hastie T (2008) A working guide to boosted regression trees. J Anim Ecol 77: 802–813. doi:10.1111/j.1365-2656.2008.01390.x
71. Thuiller W, Georges D, Engler R (2013) BIOMOD2: Ensemble platform for species distribution modeling. R package version 2.1. 7/r560.
72. Phillips SJ, Dudík M, Elith J, Graham CH, Lehmann A, et al. (2009) Sample selection bias and presence-only distribution models: implications for background and pseudo-absence data. Ecol Appl 19: 181–197.
73. Sullivan MJP, Davies RG, Reino L, Franco AMA (2012) Using dispersal information to model the species-environment relationship of spreading non-native species. Methods Ecol Evol 3: 870–879. doi:10.1111/j.2041-210X.2012.00219.x
74. Brotons L, Thuiller W, Araújo MB, Hirzel AH (2004) Presence-absence versus presence-only modelling methods for predicting bird habitat suitability. Ecography 27: 437–448.
75. Liu C, Berry PM, Dawson TP, Pearson RG (2005) Selecting thresholds of occurrence in the prediction of species distributions. Ecography 28: 385–393.
76. Liu C, White M, Newell G (2009) Measuring the accuracy of species distribution models: a review. In: Anderssen RS, Braddock RD, Newham LTH, editors. Proceedings 18th World IMACs/MODSIM Congress. Cairns, Australia. pp. 4241–4247. Available: http://www.mssanz.org.au/modsim09/J1/liu_c_J1b.pdf. Accessed 2014 April 15.
77. Allouche O, Tsoar A, Kadmon R (2006) Assessing the accuracy of species distribution models: prevalence, kappa and the true skill statistic (TSS). J Appl Ecol 43: 1223–1232. doi:10.1111/j.1365-2664.2006.01214.x
78. Franklin J, Miller J (2009) Mapping species distributions: spatial inference and prediction. Cambridge: Cambridge Univ. Press. 336 p.
79. Elith J, Leathwick JR (2009) Species Distribution Models: Ecological Explanation and Prediction Across Space and Time. Annu Rev Ecol Evol Syst 40: 677–697. doi:10.1146/annurev.ecolsys.110308.120159
80. Elith J, Ferrier S, Huettmann F, Leathwick J (2005) The evaluation strip: A new and robust method for plotting predicted responses from species distribution models. Ecol Model 186: 280–289. doi:10.1016/j.ecolmodel.2004.12.007
81. Warren DL, Glor RE, Turelli M (2008) Environmental niche equivalency versus conservatisms: Quantitative approaches to niche evolution. Evolution 62: 2868–2883. doi:10.1111/j.1558-5646.2008.00482.x
82. Warren DL, Glor RE, Turelli M (2010) ENMTools: a toolbox for comparative studies of environmental niche models. Ecography 33: 607–611. doi:10.1111/j.1600-0587.2009.06142.x
83. Mateo RG, Felicísimo ÁM, Pottier J, Guisan A, Muñoz J (2012) Do Stacked Species Distribution Models Reflect Altitudinal Diversity Patterns? PLoS ONE 7: e32586. doi:10.1371/journal.pone.0032586
84. Colwell RK (2009) Biodiversity: concepts, patterns, and measurement. Princet Guide Ecol: 257–263.
85. Pielou EC (1977) Mathematical ecology. New York: Jon Wiley & Sons. 385 p.
86. Burton ML, Samuelson LJ, Pan S (2005) Riparian woody plant diversity and forest structure along an urban-rural gradient. Urban Ecosyst 8: 93–106.
87. U.S. Fish and Wildlife Service (2012) Endangered and Threatened Wildlife and Plants; Listing 15 Species on Hawaii Island as Endangered and Designating Critical Habitat for 3 Species; Proposed Rule, 77, Federal Register 201: 63927–94018.
88. U.S. Fish and Wildlife Service (2012) Endangered and Threatened Wildlife and Plants; Endangered Status for 23 Species on Oahu and Designation of Critical Habitat for 124 Species; Final Rule, 77, Federal Register 181: 57647–57862.
89. U.S. Fish and Wildlife Service (2012) Endangered and Threatened Wildlife and Plants; Listing 38 Species on Molokai, Lanai, and Maui as Endangered and Designating Critical Habitat on Molokai, Lanai, Maui, and Kahoolawe for 135 Species; Proposed Rule, 77, Federal Register 112: 34464–34775.
90. U.S. Fish and Wildlife Service (1977) Endangered and Threatened Wildlife and Plants: Final Rule; Correction and Augmentation of Published Rulemaking, 42, Federal Register 184: 47840–47845.
91. U.S. Fish and Wildlife Service (1977) Endangered and Threatened Wildlife and Plants: Determination of Critical Habitat for Six Endagered Species, 42, Federal Register 155: 40685–40690.
92. U.S. Fish and Wildlife Service (1983) Endangered and Threatened WildlIfe and Plantt: Rule to List Panicum Carteri (Carters Panicgrass) as an Endangered Species and Determine its Critcal Habitat, 48, Federal Register, 198: 46328–46332.
93. U.S. Fish and Wildlife Service (1984) Endangered and Threatened Wildlife and Plants; Determination of Endangered Status and Critical Habitat for Kokia drynarioides (koki'o), 49, Federal Register 234: 47397–47401.
94. U.S. Fish and Wildlife Service (2001) Endangered and Threatened Wildlife and Plants; Determination of Critical Habitat for the Oahu Elepaio (Chasiempis sandwichensis ibidis), 66, Federal register 237: 63752–63782.
95. U.S. Fish and Wildlife Service (2002) Endangered and Threatened Wildlife and Plants; Designation of Critical Habitat for the Newcomb's Snail, 67, Federal Register 161: 54026–54056.
96. U.S. Fish and Wildlife Service (2003) Endangered and Threatened Wildlife and Plants; Final Designation or Nondesignation of Critical Habitat for 95 Plant Species From the Islands of Kauai and Niihau, HI; Final Rule, 68, Federal Register 39: 9116–9164.
97. U.S. Fish and Wildlife Service (2003) Endangered and Threatened Wildlife and Plants; Designation of Critical Habitat for the Blackburn's Sphinx Moth, 68, Federal Register 111: 34710–34766.
98. U.S. Fish and Wildlife Service (2003) Endangered and Threatened Wildlife and Plants; Designation of Critical Habitat for Five Plant Species From the Northwestern Hawaiian Islands, Hawaii; Final Rule, 68, Federal Register 99: 28054–28075.
99. U.S. Fish and Wildlife Service (2003) Endangered and Threatened Wildlife and Plants; Designation of Critical Habitat for the Kauai Cave Wolf Spider and Kauai Cave Amphipod; Final Rule, 68, Federal Register 68: 17430–17470.
100. U.S. Fish and Wildlife Service (2004) Endangered and Threatened Wildlife and Plants; Determination of Endangered Status and Prudency Determination for Designation of Critical Habitat for Two Plant Species From the Commonwealth of the Northern Mariana Islands, 69. Federal Register 68: 18499–18507.
101. U.S. Fish and Wildlife Service (2008) Endangered and Threatened Wildlife and Plants; Designation of Critical Habitat for 12 Species of Picture-Wing Flies From the Hawaiian Islands, 73, Federal Register 234: 73794–73895.
102. U.S. Fish and Wildlife Service (2010) Endangered and Threatened Wildlife and Plants; Determination of Endangered Status for 48 Species on Kauai and Designation of Critical Habitat; Final Rule, 75, Federal Register 70: 18960–19165.
103. Hengl T, Roudier P, Beaudette D, Pebesma E, Hengl MT (2012) Package "plotKML."
104. Lenoir J, Gegout JC, Marquet PA, de Ruffray P, Brisse H (2008) A Significant Upward Shift in Plant Species Optimum Elevation During the 20th Century. Science 320: 1768–1771. doi:10.1126/science.1156831.
105. Loope LL, Giambelluca TW (1998) Vulnerability of Island Tropical Montane Cloud Forests to Climate Change, with Special Reference to East Maui, Hawaii. Clim Change 39: 503–517. doi:10.1023/A:1005372118420
106. Daehler CC, Carino DA (2000) Predicting invasive plants: prospects for a general screening system based on current regional models. Biol Invasions 2: 93–102.
107. Daehler CC (2005) Upper-montane plant invasions in the Hawaiian Islands: Patterns and opportunities. Perspect Plant Ecol Evol Syst 7: 203–216. doi:10.1016/j.ppees.2005.08.002
108. Parmesan C (2007) Influences of species, latitudes and methodologies on estimates of phenological response to global warming. Glob Change Biol 13: 1860–1872. doi:10.1111/j.1365-2486.2007.01404.x
109. Dukes JS, Chiariello NR, Loarie SR, Field CB (2011) Strong response of an invasive plant species (*Centaurea solstitialis* L.) to global environmental changes. Ecol Appl 21: 1887–1894.

110. Asner GP, Hughes RF, Vitousek PM, Knapp DE, Kennedy-Bowdoin T, et al. (2008) Invasive plants transform the three-dimensional structure of rain forests. Proc Natl Acad Sci 105: 4519–4523.
111. Yelenik SG, D'Antonio CM (2013) Self-reinforcing impacts of plant invasions change over time. Nature 503: 517–520. doi:10.1038/nature12798.
112. Allison SD, Vitousek PM (2004) Rapid nutrient cycling in leaf litter from invasive plants in Hawai'i. Oecologia 141: 612–619. doi:10.1007/s00442-004-1679-z
113. Shiels AB (2011) Frugivory by introduced black rats (*Rattus rattus*) promotes dispersal of invasive plant seeds. Biol Invasions 13: 781–792. doi:10.1007/s10530-010-9868-7
114. Fortini LB, Price JP, Jacobi JD, Vorsino AE, Burgett JM, et al. (2013) A landscape-based assessment of climate change vulnerability for all native Hawaiian plants. Technical Report. Hilo, HI: Hawai'i Cooperative Studies Unit. University of Hawaii at Hilo. Available: http://hilo.hawaii.edu/hcsu/documents/TR44_Fortini_plant_vulnerability_assessment.pdf. Accessed 2014 April 15.
115. Peters RL, Darling JDS (1985) The Greenhouse Effect and Nature Reserves. BioScience 35: 707–717. doi:10.2307/1310052
116. Pressey RL, Cabeza M, Watts ME, Cowling RM, Wilson KA (2007) Conservation planning in a changing world. Trends Ecol Evol 22: 583–592. doi:10.1016/j.tree.2007.10.001
117. Gibbs KE, Currie DJ (2012) Protecting Endangered Species: Do the Main Legislative Tools Work? PLoS ONE 7: e35730. doi:10.1371/journal.pone.0035730
118. Owen D (2012) Critical Habitat and the Challenge of Regulating Small Harms. Fla Law Rev 64: 141.
119. Ahumada JA, Samuel MD, Duffy DC, Dobson AP, Hobbelen PH (2009) Modeling the Epidemiology of Avian Malaria and Pox. In: Pratt TK, Atkinson CT, Banko PC, Jacobi JD, Woodworth BL, editors. Conservation biology of Hawaiian forest birds: implications for island avifauna. New Haven: Yale University Press. pp. 331–355.
120. Pau S, Gillespie TW, Price JP (2009) Natural history, biogeography, and endangerment of Hawaiian dry forest trees. Biodivers Conserv 18: 3183–3183. doi:10.1007/s10531-009-9704-5
121. Mccarthy KP, Fletcher Jr RJ, Rota CT, Hutto RL (2012) Predicting Species Distributions from Samples Collected along Roadsides. Conserv Biol 26: 68–77. doi:10.1111/j.1523-1739.2011.01754.x
122. Palmer DD (2008) Hawai'i's ferns and fern allies. Honolulu: University of Hawai'i Press. 336 p.
123. Ibanez I, Clark JS, Dietze MC (2008) Evaluating the sources of potential migrant species: implications under climate change. Ecol Appl 18: 1664–1678.
124. Ibanez I, Silander Jr JA, Allen JM, Treanor SA, Wilson A (2009) Identifying hotspots for plant invasions and forecasting focal points of further spread. J Appl Ecol 46: 1219–1228. doi:10.1111/j.1365-2664.2009.01736.x
125. Silander Jr JA, Ibáñez I, Mehrhoff L (n.d.) The Biology and Ecology of Invasive Species – the Importance of International Collaboration in Predicting the Spread of Invasive Species. Proceedings of the NIAES International Symposium (Tsukuba, Japan). pp. 8–17.
126. Williams JN, Seo C, Thorne J, Nelson JK, Erwin S, et al. (2009) Using species distribution models to predict new occurrences for rare plants. Divers Distrib 15: 565–576. doi:10.1111/j.1472-4642.2009.00567.x
127. Kou X, Li Q, Liu S (2011) Quantifying Species' Range Shifts in Relation to Climate Change: A Case Study of Abies spp. in China. PLoS ONE 6: e23115. doi:10.1371/journal.pone.0023115
128. Piedallu C, Gegout J (2008) Efficient assessment of topographic solar radiation to improve plant distribution models. Agric For Meteorol 148: 1696–1706. doi:10.1016/j.agrformet.2008.06.001
